# Can AI chatbots be reliable in dental emergencies? quality assessment of Arabic responses to dental emergency inquiries and public attitudes toward their use

**DOI:** 10.1186/s12903-026-09129-1

**Published:** 2026-07-03

**Authors:** Khalid Talal Aboalshamat, Abrar Khalid Demyati, Abdalmalik Osamah Ghandourah, Shumukh Yousef Balhmer, Wareef Omar Ghazzawi, Sali Abdullah Sayed, Refal Abdullah Aljabri, Rama Juwaybir Alhuzali

**Affiliations:** 1 Dental Public Health Division, Preventative Dentistry Department, College of Dental Medicine, Umm Al-Qura University, Makkah, Saudi Arabia; 2 Oral Maxillofacial Surgery Department, College of Dental Medicine, Umm Al-Qura University, Makkah, Saudi Arabia; 3 College of Dental Medicine, Umm Al-Qura University, Makkah, Saudi Arabia

**Keywords:** Artificial intelligence, Chatbots, Dental emergencies, Public attitudes, Arabic language, ChatGPT

## Abstract

**Background:**

Artificial intelligence (AI) is increasingly penetrating health and dental fields without sufficient monitoring of its quality and applicability.

**Aim:**

This study aimed to evaluate public attitudes toward AI chatbots in dental emergencies and assess the quality of Arabic-language responses generated by different AI chatbots for dental emergency inquiries.

**Methods:**

The study had two parts. Part one: A cross-sectional online survey where 441 Saudi residents aged ≥ 18 years answered a 33-item questionnaire in Arabic that included 14 items to measure attitudes about the use of AI chatbots in dental emergencies with a 5-point Likert scale. Part two: From participant answers and oral and maxillofacial surgeons, we selected 50 dental inquiries about dental emergencies and presented them in Arabic to five AI chatbots (ChatGPT-5.1, Google Gemini 3, Claude Sonnet 4.5, Grok 1.3.40, and DeepSeek 3.2). Responses were evaluated by two calibrated oral and maxillofacial surgeons using 5-point Likert scales for accuracy, clarity, comprehensiveness, relevance, and acceptability.

**Results:**

Participants showed moderately positive attitudes (2.72–3.89/5) about AI chatbots for dental emergencies. AI chatbots had generally high mean scores for accuracy (4.08–4.87), clarity (4.21–4.92), comprehensiveness (4.10–4.67), relevance (4.11–4.91), and acceptance (3.84–4.89). No significant differences were found among the AI chatbots, except Grok, which scored lower than the others on multiple quality measures (all *p* < 0.001). Inter-rater reliability varied across chatbots, single-measure ICC values ranging from 0.23 to 0.60; however, exact agreement was 69.8%, and 94.5% of paired ratings differed by no more than one point.

**Conclusion:**

Saudi public attitudes toward AI chatbots in dental emergencies were moderate. Overall, the quality of Arabic AI chatbot responses was high, although Grok had significantly lower ratings. Human supervision remains essential, and continuous “living” evaluations are needed to track rapidly evolving chatbot performance.

**Supplementary Information:**

The online version contains supplementary material available at 10.1186/s12903-026-09129-1.

## Background

Artificial Intelligence (AI) involves enabling machines to replicate human cognitive abilities to perform tasks that normally require human intelligence [1]. An AI chatbot is an advanced program that uses machine learning and natural language processing (NLP) to engage with users in a conversation resembling human interaction [2, 3]. There are several AI chatbots, including ChatGPT (OpenAI), Gemini (Google), Claude (Anthropic), Grok (xAI), and DeepSeek (DeepSeek AI). AI chatbots are evolving at a rapid pace, and there is continuous improvement and expansion of the large language models used by each AI chatbot [4, 5]. Newer AI chatbot models often exhibit improved reasoning, accuracy, and task performance, but may also increase computational cost and response time. Therefore, companies provide multiple models to efficiently suit different tasks. The adoption of AI in dentistry has been beneficial for applications such as diagnostic imaging [6, 7], diagnosis and treatment planning [8], caries detection [9, 10], and dental information retrieval [11].

Attitudes about AI in dentistry have been investigated in multiple studies [11–15]. In fact, dental professionals and patients show generally optimistic views of AI [12, 14], and its use has become more acceptable among dental students and dentists [16, 17]. Kostov and Yordanova (2025) found moderate attitudes about AI chatbot use in dentistry among dentists, dental students, and patients when applied as an adjunct tool, but not for sole diagnosis [13]. However, these studies showed mixed in-depth details. In Saudi Arabia, healthcare workers exhibited mixed views about ChatGPT: 18.4% had used it, 84.1% were interested in future use, 65.9% found it helpful for medical research, and 39.5% considered it useful for decision-making [15]. However, 46.9% had credibility concerns, and 30.8% were worried about medicolegal implications [15]. Among the general public, 33.7% had used ChatGPT previously, but only 8.7% had used it for dental purposes; overall attitudes were moderate, with lower rates of agreement that AI chatbots could replace human interaction [11].

Several studies investigated the quality and validity of AI chatbots as a source of dental information and found it to be of moderate to high quality [18, 19], but with difficulty in readability requiring a higher education level, making it challenging to understand for the general public [19]. The majority of recent studies found AI chatbots used by the general public to have moderate to high rates of accuracy [11, 20–23]. Reported accuracy or correctness ranged from 71.3% positive ratings for ChatGPT-4o in photo-based intraoral trauma assessment to 95.6% accuracy for paid ChatGPT-4 in avulsion injury management [20, 22]. In traumatic dental injury queries, other studies have reported overall correctness scores of 96.61% for ChatGPT-4, 94.91% for Gemini, and 81.35% for ChatGPT-3.5 [21]. One Arabic study reported a mean accuracy score of 4.67 out of 5 for ChatGPT-4o mini instead of a percentage-based accuracy value [11].

Additionally, substantial differences exist across studies concerning topics, AI models, question types, and scoring systems. Question topics vary between dental avulsion management [22, 24], diagnosis [20], and dental trauma patient queries [21]. Some studies evaluated a single AI model [11], whereas others compared multiple models [20–24]. Most assessed dental specialist questions [21–23], whereas others used questions from the general public [11, 20, 24]. Scoring methods also differed, ranging from true/false scoring [22] to Likert scales [11, 21, 23, 24]. Therefore, the overall quality of the evidence is difficult to judge. One of the notable aspects seen in reviewing previous studies is that there is variation in the AI chatbot models used, with some studies even failing to report some of the AI chatbot models used [20–22], which complicates validity judgement.

Dental emergencies often require timely and understandable guidance, and members of the public may increasingly seek urgent health information from AI chatbots. Previous work has highlighted the importance of evaluating chatbot responses in urgent dental contexts, including emergency and trauma-related inquiries [25]. However, evidence remains limited on the quality of Arabic AI chatbot responses to dental emergency questions and on public acceptance of using these tools in such situations. This is particularly relevant in Saudi Arabia, where Arabic is the main language of communication and linguistic clarity may influence users’ understanding and trust.

## Aim

This study aimed to evaluate public attitudes about the use of AI chatbots in dental emergencies and assess the quality of responses from different AI chatbots to dental emergency inquiries in the Arabic language.

### Methods

This study was conducted in two parts. The first part assessed the attitudes about AI chatbot use among the public with regard to dental emergencies. The second part assessed the quality of responses from different AI chatbots to queries about dental emergencies.

### Part 1

#### Participants

A convenience sampling approach was applied to recruit Saudi residents aged 18 years and older. Participants were excluded if they lacked internet access or were unable to read and write. For this study, the sample size was calculated to be at least 385 participants, based on a 50% assumed prevalence, 5% margin of error, and 95% confidence level. The sample size was derived using the equation *n* = (Z² × P × (1 − P)) / d², where Z represents the Z-score for the chosen confidence level (1.645), P is the assumed prevalence (50%), and d indicates the margin of error (0.05). Participants were recruited via an online survey distributed through various social media platforms, including Telegram, Instagram, Snapchat, WhatsApp, and X (formerly known as Twitter). Invitations were distributed by the research team through participants’ personal accounts and public social media groups, in addition to direct personal invitations. The study was submitted for approval to the institutional review board of Umm Al-Qura University, Makkah, Saudi Arabia, prior to data collection (HAPO-02-K-012-2025-10-2960). The questionnaire was administered anonymously, and any information that could potentially reveal participants’ identities was removed prior to analysis.

#### Questionnaire

The study questionnaire was sent to participants in electronic form in Arabic, and permission was obtained when the “Next” button on the first page of the questionnaire was clicked. Participants who refused to click “Next” were excluded from the study. Each participant was given a unique identification number to protect confidentiality and to maintain participant anonymity.

The questionnaire consisted of four sections with a total of 33 questions. The first section collected 9 demographic items, including age, nationality, region of residence, education level, prior experience with dental crises, and emergency cases. Following that section, a small definition of AI chatbot was provided, along with an illustrated picture (Fig. i1).

**Fig. 1:**
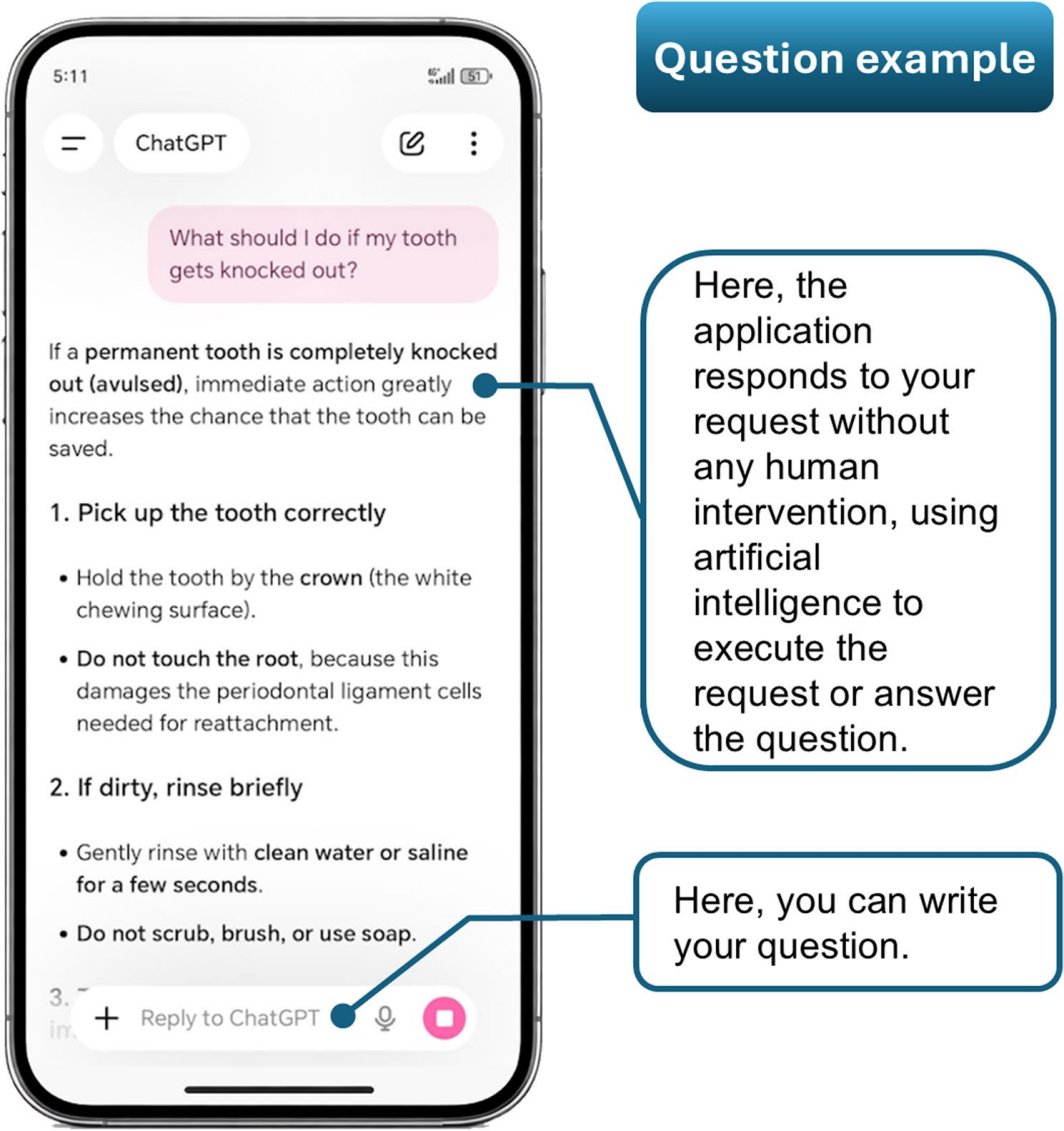
AI Chatbot illustration (English-translated) provided to participants to demonstrate how it works

The second section of the questionnaire contained 9 questions to explore the usability of different AI chatbot platforms. This section included whether they had used AI chatbots in general contexts or specifically for obtaining medical, dental, or dental emergency related information. Furthermore, this section included questions about the specific chatbot applications that participants had previously used. The third section of the questionnaire contained 14 questions and focused on AI chatbots. These questions captured participants’ attitudes about AI chatbots (trust, safety, usefulness, and willingness to rely upon them) in dental emergency situations. The participants provided closed-ended answers. A 5-point Likert scale was used to record the responses (1 = strongly disagree to 5 = strongly agree). These questions were derived from a previous study that assessed the attitudes of pregnant women toward ChatGPT in the dental field, with modifications [11]. Those modifications included changing the situation from pregnancy in a dental setting to emergencies in a dental setting. The fourth section included an optional open question asking participants to write three dental questions of their choosing that they wanted to ask an AI chatbot. The questionnaire went into pilot round for face validation, to check grammar, syntax, context, content, logic and flow for 10 individuals. The results of those individuals were not included in the main study results.

### Part 2

#### Question selection process: source, exclusions, and inclusions

In the second part of the study, the questions taken from the participants in the fourth section of the first part were collected, organized, categorized according to emergency themes and reviewed. Questions unrelated to dentistry, unrelated to dental emergencies, or duplicated were excluded. Additional questions were then added by the oral and maxillofacial surgery consultants based on emergency inquiries commonly encountered in clinical practice. The final selection was made by the two consultants and the research team, based mainly on clinical importance and frequency in dental emergency settings, with greater weight given to the consultants’ clinical judgment. The final 50 Arabic questions were categorized according to the emergency topic and are provided as English translation in Supplementary File S2. The questions were designed to reflect common urgent dental conditions and patient concerns, and measures were taken to maximize the diversity of questions.

These questions were subsequently presented in Arabic to the latest free model of five AI chatbots during the AI chatbot data collection phase (November 20–29, 2025), which included ChatGPT-5.1, Google Gemini 3, Claude (Sonnet 4.5), Grok 1.3.40, and DeepSeek 3.2. All 50 questions were submitted sequentially within a single chat session for each AI chatbot, using identical Arabic questions across AI chatbots. Each chatbot generated responses in Arabic, which were then collected for further evaluation of their quality using predetermined criteria that focused on accuracy, clarity, comprehensiveness, relevance, and acceptance. These measurements were taken from previous studies [11, 26, 27].

#### AI chatbot setup and procedure

For each AI chatbot, a new email account was created to mitigate potential biases arising from prior account history or personalization effects. Access to the five models was initiated via Google search, with authentication completed using the “Continue with Google” option. Google was chosen as the entry point in order to reflect a typical user experience, given its prominence as the most widely utilized search engine.

A designated team member submitted each question to the corresponding AI chatbot, and the resulting responses were compiled into Word documents for comparison. Because of daily query limits enforced by the AI chatbots, data collection was spread across six consecutive days. To maintain uniformity, all queries were issued at the same time each evening (9:30 p.m.). Different members of the research team were designated to specific AI chatbots.

Prior to initiating queries on each AI chatbot using official AI chatbot Apps, browsing history and cookies were cleared through the browser’s privacy and security settings. The selected options for clearing included cached images and files, cookies, other site data, and browsing history. This procedure ensured a neutral and standardized testing environment across all five AI chatbots.

#### Quality assessment of AI chatbot answers

Each response generated by ChatGPT-5.1, Google Gemini 3, Claude (Sonnet 4.5), Grok 1.3.40, and DeepSeek 3.2 to dental emergency questions was evaluated by two senior oral and maxillofacial surgeons. The two surgeons were calibrated via 6 h of section training conducted through a 2-day workshop. The calibration workshop included 10 AI-generated responses to general dental questions. Each evaluator rated the responses independently, after which the scores were compared and discussed. Any differences were resolved through discussion to clarify the interpretation of each rating criterion before the full evaluation began. The assessment followed a 5-point Likert scale (1 = strongly disagree to 5 = strongly agree) adapted from previous studies [11, 28]. The evaluation criteria included: (1) accuracy: alignment of the response with dental literature and scientific guidelines; (2) clarity: ease of understanding; (3) comprehensiveness: extent to which all aspects of the question were addressed; (4) relevance: directness in addressing the query; and (5) acceptance: suitability of the response without requiring modifications. Although the AI-generated responses varied in length and depth, each sentence was reviewed individually. The information in the responses was categorized as commonly known by general dentists, questionable, or seemingly incorrect. Any content identified as questionable or potentially incorrect by either assessor was color coded for further review. The accuracy of the evaluated content was verified using established dental literature and clinical practice guidelines. Statements that conflicted with evidence-based findings were re-examined and discussed by the research team to reach a consensus. This consensus process was used to resolve the scientific interpretation of questionable or incorrect content, while the original evaluator scores were retained for quantitative analysis. However, it should be stated that in the main evaluation, each consultant scored the AI chatbot responses independently. The final quantitative score for each item was calculated as the mean of the two evaluators’ scores, rather than replacing their ratings with a consensus score. This was preserved to give each evaluator, their own judgement and scoring even after calibration.

The AI chatbot responses were assessed in terms of readability using a freely available readability calculator [29]. For each response, the following measures were recorded: number of lefters, number of sentences, Flesch–Kincaid grade level (FKGL), simple measure of gobbledygook (SMOG), and Flesch reading ease (FRE). Based on previous studies [30, 31], An FKGL score of 7 indicates that the text can be understood by a reader with an approximately seventh-grade education level, and a SMOG score of 7 indicates that about 7 years of education are needed to understand the text. An FRE score of 80 or higher indicates text that is easy to read and understandable for most readers.

#### Data analysis for part 1 and part 2

Statistical analysis was conducted using SPSS software version 31 (IBM Corp., Armonk, NY, USA) and Microsoft Excel. In addition to descriptive statistical methods, the statistical analyses included two-way random-effects intraclass correlation coefficient (ICC) for inter-rater reliability, Cronbach’s alpha for internal consistency. Exact percentage agreement was calculated by dividing the number of identical ratings between the two evaluators by the total number of paired ratings. Agreement within one point was also calculated, meaning that ratings were considered close if the two evaluators’ scores differed by no more than one point on the 5-point Likert scale. Statistical tests used included one-way ANOVA with Tukey post hoc tests for chatbot quality-score comparisons, and Kruskal–Wallis tests for readability comparisons. The results were evaluated at a significance level of *P* < 0.05.

## Results

### Part I: attitudes about AI chatbots in dental emergencies

This study included 441 participants, with a mean age of 31.27 and standard deviation (SD) of 13.42. Participants’ demographic data are presented in (Table 1).

**Table 1:** Participant demographic data (*N* = 441)

Variable		*n*	%
Gender	Male	80	18.14%
	Female	361	81.86%
Nationality	Saudi	408	92.52%
	Non-Saudi	33	7.48%
Region	Western	345	78.23%
	Central	53	12.02%
	Eastern	22	4.99%
	Southern	13	2.95%
	Northern	8	1.81%
Education level	Less than bachelor’s	84	19.05%
	Bachelor’s degree	326	73.92%
	Higher education	31	7.03%
Are you in a dentistry or healthcare field?	Yes	133	30.16%
	No	308	69.84%
English level	I do not know English	19	4.31%
	Beginner	86	19.50%
	Intermediate	255	57.82%
	High level	81	18.37%
Have you ever had a dental emergency, such as toothache, severe pain, or a broken tooth?	Yes	257	58.28%
	No	184	41.72%
Have you ever been to a hospital or dental clinic due to an emergency or pain?	Yes	317	71.88%
	No	124	28.12%

Participants' awareness of AI chatbots and their previous use in the dental field are displayed in (Table 2). Participants had differing usage of various AI chatbot platforms. For example, there were 400 (90.70%) who had used ChatGPT, 146 (33.10%) who had used Gemini, 66 (14.96%) who had used Grok, 65 (14.73%) who had used DeepSeek, and 39 (8.84%) who had used Claude.

**Table 2:** Participants' awareness of AI chatbots and previous use in dental field (*N* = 441)

Statement	Yes *n* (%)	No *n* (%)
Do you know what AI chatbot applications are (such as ChatGPT)?	412 (93.42%)	29 (6.58%)
Have you ever used AI chatbot applications in general?	407 (92.29%)	34 (7.71%)
Have you ever used AI chatbot applications to get dental information in general?	179 (40.59%)	262 (59.41%)
Have you ever used AI chatbot applications to get dental emergency information?	128 (29.02%)	313 (70.98%)

The participants’ attitudes about AI chatbots with regard to dental emergencies are shown in (Table 3). The 14 attitude questions had a Cronbach’s alpha of 0.94. A multiple linear regression with backward elimination was conducted to identify factors associated with the total attitude score. The total attitude score ranged from 14 to 70, with a mean of 47.47 ± 11.32. In the final model, gender was the only retained significant predictor, F(1,439) = 19.94, *p* < 0.001, explaining 4.3% of the variance in total attitude score, R² = 0.043. Since gender was coded as male = 1 and female = 2, the negative coefficient indicates that female participants had lower total attitude scores than male participants, B = -6.12, SE = 1.37, β = -0.208, *p* < 0.001. Descriptively, males had a higher mean total attitude score (m = 52.48, SD = 11.77) than females (m = 46.36, SD = 10.93). Other variables, including age, nationality, region, education level, healthcare/dentistry background, and awareness of AI chatbot applications, were removed during backward elimination and were not retained in the final model. The final model explained 4.3% of the variance in total attitude score (R² = 0.043).

**Table 3:** Participant attitudes about AI chatbots in relation to dental emergencies

Statement	m (SD)
AI chatbot apps are competent at providing information and guidance related to dental emergencies.	3.71 (0.99)
AI chatbot apps are reliable and consistent in offering dependable information about dental emergencies.	3.35 (1.04)
AI chatbot apps are transparent in providing information about dental emergencies.	3.89 (0.90)
AI chatbot apps are trustworthy when answering questions about dental emergencies.	3.28 (1.06)
AI chatbot apps do not manipulate the responses to questions related to dental emergencies.	3.27 (1.11)
AI chatbot apps are secure and protect my privacy and confidential information when answering my questions about dental emergencies.	3.05 (1.17)
I am willing to use AI chatbot apps for inquiries related to dental emergencies.	3.41 (1.14)
The benefits of using AI chatbot apps outweigh any potential risks associated with dental emergency inquiries.	3.28 (1.08)
AI chatbot apps help me make informed and timely decisions about dental emergencies.	3.52 (1.00)
I am willing to make decisions based on recommendations provided by AI chatbots regarding dental emergencies.	3.21 (1.13)
I am satisfied with AI chatbot apps as a source of dental emergency related assistance.	3.84 (0.92)
I am willing to use AI chatbot apps in the future regarding dental emergencies.	3.26 (1.20)
AI chatbot apps can replace human interaction for dental emergency inquiries.	2.72 (1.34)
AI chatbots are persuasive when it comes to providing answers about dental emergencies.	3.67 (0.95)

### Part 2: accuracy of AI chatbots in dental emergencies

In this portion of the study, two expert evaluators measured the accuracy of AI chatbot responses to assess accuracy, clarity, comprehensiveness, relevance, and acceptance. Inter-rater reliability assessed using a two-way random-effects intraclass correlation coefficient (ICC) for consistency showed moderate agreement for ChatGPT (ICC = 0.55) and DeepSeek (ICC = 0.60), while Claude (ICC = 0.31), Grok (ICC = 0.30), and Gemini (ICC = 0.23) demonstrated low agreement at the single-measure level. In contrast, Cronbach’s alpha values indicated high to very high internal consistency across AI chatbots (ChatGPT α = 0.93; Claude α = 0.82; Grok α = 0.81; DeepSeek α = 0.94; Gemini α = 0.74) and overall (α = 0.86). Overall, the two evaluators gave identical ratings in 872 out of 1,250 paired ratings, resulting in an exact percentage agreement of 69.8%. When allowing a one-point difference on the 5-point Likert scale, agreement increased to 1,181 out of 1,250 paired ratings, or 94.5%. Cronbach’s alpha values were also reported to describe the internal consistency of the rating items; however, these values were interpreted separately from ICC because they do not measure inter-rater agreement.

After taking the means of both evaluators, the quality of different AI chatbot answers to queries about dental emergencies is shown in (Table 4). Using analysis of variance (ANOVA; Tukey post hoc), Grok scored significantly lower than all other AI chatbots for accuracy, clarity, relevance, and acceptance (all *p* < 0.001). For clarity, DeepSeek was also significantly lower than Gemini (*p* = 0.021). For comprehensiveness, Grok was significantly lower than DeepSeek (*p* = 0.040) and Gemini (*p* < 0.001).

**Table 4:** Comparison of evaluators’ mean quality scores for the different AI chatbots’ answers

AI chatbot	Accuracy m (SD)	Clarity m (SD)	Comprehensiveness m (SD)	Relevance m (SD)	Acceptance m (SD)
ChatGPT-5.1	4.60 (0.60)*	4.78 (0.41)*	4.34 (0.67)	4.91 (0.33)*	4.61 (0.66)*
Claude (Sonnet 4.5)	4.67 (0.50)*	4.91 (0.19)*	4.35 (0.71)	4.90 (0.42)*	4.64 (0.54)*
Grok 1.3.40	4.08 (0.59)	4.21 (0.66)	4.10 (0.65)	4.11 (0.74)	3.84 (0.75)
DeepSeek 3.2	4.62 (0.71)*	4.66 (0.48)^$^	4.47 (0.75)^#^	4.70 (0.73)*	4.59 (0.81)*
Gemini 3	4.87 (0.32)*	4.92 (0.21)^$^	4.67 (0.45)^#^	4.83 (0.48)*	4.89 (0.32)*

* Significantly higher than Grok for the indicated outcome (all p < 0.001)

^$^Gemini significantly higher than DeepSeek for clarity (*p *= 0.021)

^#^DeepSeek and Gemini significantly higher than Grok for comprehensiveness (DeepSeek:*p *= 0.040; Gemini:*p* < 0.001)

In terms of readability measures for the five AI chatbots, the number of characters, sentences, FKGL, SMOG, and FRE are shown in Table 5, determined by using the readability calculator. The median and inter-quartile ranges were used because the data showed no normal distribution. FKGL estimates the grade level required for comprehension based on sentence length and syllables, and an FKGL score < 7 indicates text below a seventh-grade level. All AI chatbots were below this threshold (0.94–6.67), with DeepSeek lowest (0.94) and ChatGPT highest (6.67). SMOG estimates years of education required based on polysyllabic words and vocabulary complexity, and a SMOG score < 7 indicates lower educational demand. All of the AI chatbots were below the cutoff (3.00–4.78); Claude and DeepSeek were lowest (3.00), and Grok was highest (4.78). In FRE measures, ≥ 80 should be classified as easy. All AI chatbots exceeded this threshold (94.71–109.31), with DeepSeek highest (109.31) and ChatGPT lowest (94.71). All of the AI chatbots met easy readability standards, with DeepSeek demonstrating the simplest profile across indices but with the highest number of characters and sentences.

**Table 5:** AI chatbot answers to dental emergency inquiries: number of characters, sentences, FKGL, SMOG, and FRE

Variable	ChatGPT Median (Q1–Q3)	Claude Median (Q1–Q3)	Grok Median (Q1–Q3)	DeepSeek Median (Q1–Q3)	Gemini Median (Q1–Q3)
Number of characters	844.5 (735–1010)	622.5 (485–722)	1340 (1094–1558)	1626.5 (1415–1878)	1363.5 (1207.5–1583)
Number of sentences	7.5 (6–10)	6 (5–8)	20 (14–25)	30.5 (26–38)	15.5 (14–20)
FKGL	6.67 (3.58–9.41)	6.32 (4.67–8.68)	2.53 (2.03–4.35)	0.94 (0.61–1.49)	3.81 (2.78–4.69)
SMOG	3.00 (3.00–5.45)	3.00 (3.00–3.00)	4.78 (3.86–5.54)	3.00 (3.00–4.07)	4.27 (3.00–5.24)
FRE	94.71 (86.87–102.99)	95.85 (89.57–100.16)	103.20 (98.94–106.58)	109.31 (108.18–110.56)	101.50 (98.97–104.50)

FKGL: Flesch–Kincaid grade level

SMOG: Simple measure of gobbledygook

FRE: Flesch reading ease

The Kruskal–Wallis test demonstrated statistically significant differences between AI chatbots for all examined variables, including number of characters (χ²(4) = 166.99, *p* < 0.001), number of sentences (χ²(4) = 182.99, *p* < 0.001), FKGL (χ²(4) = 119.51, *p* < 0.001), SMOG (χ²(4) = 40.01, *p* < 0.001), and FRE (χ²(4) = 110.28, *p* < 0.001).

## Discussion

This study assessed public attitudes about AI chatbots in Saudi Arabia and the quality of responses of different AI chatbots to dental emergency inquiries in Arabic. Participants reported generally moderate positive attitudes toward AI chatbots being used for dental emergency related inquiries. Expert evaluations of AI chatbots showed that responses were rated highly for accuracy, clarity, comprehensiveness, relevance, and acceptance. However, Grok was consistently significantly lower than other AI chatbots in most of the domains.

Awareness about the use of AI chatbots in Saudi Arabia has varied widely, ranging from 42% [11] and 49.1% [15] to 90% [32], which is lower than the awareness reported in our results (93.42%). In fact, this suggests that awareness about AI chatbots and their use in health-related aspects are growing across years in local studies in Saudi Arabia, consistent with international findings [33, 34].

Attitudes about use of AI chatbots in healthcare and education varies [11, 15, 32, 35, 36]. A recent study found generally positive attitudes among the Saudi public toward using ChatGPT for dental inquiries in terms of competency, future use, and transparency, and moderate attitudes regarding a belief in the abilities of AI to replace human interaction [11], which is similar to our findings, which measured the same outcomes. This positive attitude is supported by previous studies [15, 32, 35], with some indicating that 76.7% of Saudi healthcare workers believed ChatGPT could positively impact healthcare, and 81.8% wanted to learn more about it [15]. Most of these studies focused on ChatGPT, whereas our results extrapolate the positive attitudes to other AI chatbots (Claude, DeepSeek, Gemini, Grok) that are in a very competitive race to lead the generative AI scene [37].

One of the findings that male had higher positive attitude toward using AI chatbots in dental emergency than female, while other variables have no influence. However, the sample was imbalanced by gender and region, with females and participants from the Western region forming the majority. This may have influenced the overall attitude findings and should be considered when interpreting their generalizability.

Several studies have evaluated the quality of AI chatbot responses in the dental field. Some assessed the quality of the contents of responses to inquiries about dental trauma or avulsion and pain management [20–24], which are the most similar to our selected topic of dental emergency. The quality assessments included measuring the constructs of accuracy, completeness, clarity, relevance, or a combination thereof [11, 21, 24]. These constructs were used as the basis for the following comparisons.

Overall, the accuracy of AI chatbots in our study had a mean of 4.08 to 4.87 out of 5, indicating high levels of accuracy. This was similar to the majority of the recent studies of AI chatbot responses to dental questions [11, 20–23]. Some studies measured accuracy on a scale of five points, similar to our study, and reported that the accuracy of ChatGPT-4o answers to dental related questions ranged from 4.67 to 4.74 out of 5 [11, 23]. This high level of accuracy of AI chatbot responses is supported by other studies that measured accuracy and correctness by percentages, with accuracy of 95.6% for ChatGPT-4 (paid model) [22] and 81.35% to 96.61% for different ChatGPT models [21], . Nevertheless, our results are contradictory to two previous studies [24, 38]. One found the accuracy to be 51% and 64% for ChatGPT-3.5 and Google Bard (the prior name for Gemini), respectively [38]. This can be explained by the use of an older model (ChatGPT-3.5) [38] in that study, as compared to our use of recent models. The other study reported the accuracy to be 6.05 out of 10 for Claude [24]. However, that study assessed AI chatbot responses regarding management of acute dental pain, which might contain specific dosage information that can be sensitive to error [24]. A patient-safety concern was observed in one Grok response about taking antibiotics for pulpitis without visiting a dentist for question 35. Although Grok generally discouraged unsupervised antibiotic use, it provided specific antibiotic options and doses if dental care was unavailable within 24 h. This may encourage self-medication and highlights the need for professional supervision when AI chatbots provide dental emergency advice. Thus, AI chatbot responses to dental related questions are likely to have high levels of accuracy, but they are not perfect, and human supervision is needed.

When comparing the accuracy of the AI chatbots and their models, our results showed no significant difference between models except for Grok. However, most trauma-focused studies have ranked the ChatGPT-4 series as most accurate [21–23], while another found Claude to have higher rates of accuracy [24]. However, previous studies have typically examined fewer chatbots or focused mainly on ChatGPT. In contrast, we compared five recent AI chatbot models simultaneously, and the differences found were small and not significant. Nevertheless, Grok showed relatively lower accuracy, which is new to the literature, as Grok responses to dental questions have not been investigated in the dental literature sufficiently to date. It is crucial to mention that many studies did not report the exact models of Claude, Perplexity, Gemini, or Copilot [20–22, 24], whereas ChatGPT models were usually specified [11, 21–24], which further complicates validity judgments. Differences in accuracy rates between studies may be related to AI chatbot platform, models, nature of the topic investigated, or scoring systems. Future studies might consider these points in comparison and investigate response consistency over time, as investigated in previous studies [20, 22].

Regarding comprehensiveness, clarity, and relevance, some studies [11, 23] reported results similar to ours. Aboalshamat et al. found very high means for comprehensiveness (4.87/5), clarity (4.95/5), and relevance (4.90/5) [11], closely aligning with our highest values (4.67/5, 4.92/5, and 4.91/5, respectively). Sezer and Aydoğdu also reported high levels of completeness (2.78/3) [23], proportionally comparable to a strong 5-point performance in our study. In contrast, Tosun and Öztürk reported lower scores for AI chatbot comprehensiveness (6.30/10), clarity (6.20/10), and relevance (6.80/10) [24], which may be explained by the previously discussed factors. This again suggests that AI chatbots return high-quality responses in the majority of studies, but they are, again, not flawless. This should be noted, especially when assessing responses that might jeopardize user health.

All AI chatbots in our results showed low readability demand, indicating below seventh-grade, easy text based on readability measures (FRE, SMOG, and FKGL). That contradicts previous studies that found AI chatbot responses to have more complexity, reflecting high school and university levels [23] of education and greater educational demand [21] using the same readability measures. This can be explained by the fact that the previous studies investigated responses to specialized questions posed by dental practitioners in English [21, 23] versus a study that assessed responses to public questions in Arabic. In fact, our study is one of the few studies assessing Arabic responses from AI chatbots [11].

Interestingly, DeepSeek showed the lowest readability demand (lowest FKGL and SMOG, highest FRE) despite having the highest character count, while ChatGPT showed the highest readability demand. This finding may inform the selection of the suitable AI chatbot for content generation in dental promotional campaigns for Arabic-speaking audiences, based on the needed outcome. Furthermore, our results suggest that DeepSeek used longer responses to explain information in a simpler and more accessible manner. However, this cannot be confirmed because the result may also reflect limitations of automated English-based readability formulas when applied to Arabic text. Future qualitative studies should assess Arabic-speaking users’ understanding, perceived clarity, and interpretation of AI chatbot responses. It should be noted that FKGL, SMOG, and FRE were originally developed and validated for English text. Therefore, their application to Arabic chatbot responses may not fully reflect Arabic readability, and these findings should be interpreted with caution. In fact, language differences are critical, as AI chatbots and large language models may perform differently across different languages, yielding unexpected results [39, 40].

Grok generated culturally influenced responses that were not observed in the other chatbots, including a reference to the “evil eye” and occasional sarcastic phrasing. Although such expressions may reflect familiar cultural language, their use in dental emergency communication may reduce clinical seriousness, introduce ambiguity, or affect patient trust. This raises a cultural-safety concern, as health advice should remain clear, respectful, and clinically focused across linguistic and cultural contexts. The reason for this pattern cannot be determined from our data and may relate to differences in model design, alignment, or conversational style. Future studies should further examine cultural appropriateness, tone, and safety in Arabic AI-generated health communication. The findings of this study support the growing AI-dentistry evidence showing that chatbot performance varies across dental contexts and still requires professional oversight before patient-facing use [41, 42].

This study is among the few studies that concurrently evaluated multiple generative AI chatbot platforms in response to questions from the public. It is also one of the few assessing Arabic responses, addressing potential linguistic differences than with the use of English- or Chinese-trained models. However, one of the drawbacks of our study is that the examiners’ inter-rater reliability was not optimal, despite calibration efforts. Although the single-measure ICC values indicated low to moderate inter-rater agreement for some chatbots, the overall exact percentage agreement was 69.8%, and 94.5% of paired ratings differed by no more than one point on the 5-point Likert scale. This suggests that most discrepancies were minor in magnitude. Nevertheless, the low ICC values indicate variability in evaluator scoring and should be considered when interpreting the precision of the quality scores. Also, the measurement tool, despite previous usage in many studies, lacked standardized rating thresholds, introducing potential subjectivity. Evaluators were not blinded to the AI chatbot name, which may have introduced bias. Because questions were submitted sequentially within one session, later responses may have been influenced by prior context.

One of the major limitations in this study is that the rapid evolution of AI models challenges validity, and findings may quickly become outdated despite prompt publication, limiting traditional cross-sectional approaches. Between the time of data collection and manuscript writing, ChatGPT and other AI chatbots produced multiple new models and versions that might work differently than the model examined in this study. Future research should utilize ongoing live evaluations of AI chatbots and their emerging models to create an ongoing assessment of AI chatbot responses, particularly with regard to health and dental related topics. A conventional research time frame cannot adequately align with the rapid evolution of AI and AI chatbot models.

## Conclusion

The public respondents in Saudi Arabia expressed moderate attitudes about the use of AI chatbots in dental emergency contexts, with variability across attitude domains. The quality of all AI chatbots was high, but Grok had significantly lower performance in answering dental emergency related inquiries. The use of AI for dental emergencies and health consultation needs to be supervised by human practitioners. More living studies are needed to capture the quality of AI chatbots in dentistry to cope with the rapid evolution of AI chatbot model and the emergence of new ones in order to provide a concurrent recommendation for public use.

## Supplementary Information

Supplementary Material 1.

Supplementary Material 2.

## Data Availability

The supplemental file for this paper S1 contains all the data produced or analyzed during this investigation. The supplemental file S2 contained all the questions used in the AI chatbots.

## Abbreviations

AI: Artificial intelligence
FKGL: Flesch–Kincaid grade level
FRE: Flesch reading ease
SD: Standard deviation
SMOG: Simple measure of gobbledygook
