# Supplementary material for: Can AI chatbots be reliable in dental emergencies? quality assessment of Arabic responses to dental emergency inquiries and public attitudes toward their use
Source: BMC Oral Health. 2026 Jul 3;26:1476. doi: 10.1186/s12903-026-09129-1 (PMC13479445; doi:10.1186/s12903-026-09129-1)
Supplement: Supplementary file 1 [file 12903_2026_9129_MOESM1_ESM.pdf]

SN	Age	Gender	Nationality	Region	Education	Filed	English_level	Emergency	Emergency2	A1
1	31	2	1	1	2	1	3	1	1	1
2	23	2	1	1	2	1	3	1	1	1
3	29	1	1	2	2	2	3	1	1	1
4	24	2	1	1	2	1	4	2	2	1
5	24	1	1	2	2	2	4	2	2	1
6	22	2	1	2	2	1	4	1	1	1
7	21	2	1	2	2	2	2	1	2	1
8	26	1	1	1	2	2	2	1	1	1
9	20	2	1	1	1	2	3	1	2	1
10	23	2	1	5	1	1	3	1	1	1
11	24	1	1	1	2	1	3	2	1	1
12	20	2	1	1	1	2	3	2	2	1
13	26	1	1	2	1	2	3	1	1	1
14	25	1	1	5	2	1	3	1	1	1
15	21	2	1	5	1	2	2	2	2	1
16	24	1	1	1	2	2	4	2	2	1
17	45	2	1	1	2	1	3	1	1	1
18	19	2	1	1	2	1	4	2	1	1
19	35	2	1	1	2	2	2	2	2	1
20	55	2	1	1	2	2	2	1	1	1
21	30	2	1	1	2	2	4	2	2	1
22	22	2	1	1	2	2	3	1	1	1
23	56	2	1	1	2	2	3	1	1	1
24	50	2	1	1	2	2	1	1	1	1
25	20	2	1	3	1	2	2	1	1	1
26	22	2	1	1	2	1	4	2	2	1
27	64	2	1	1	2	2	3	1	1	1
28	41	2	1	1	2	2	3	2	1	1
29	30	2	1	1	2	2	3	2	2	1
30	40	2	1	1	2	2	3	1	1	1
31	23	2	1	1	2	1	3	1	1	1
32	18	2	1	1	1	2	3	2	2	1
33	27	1	1	3	2	2	3	1	1	1
34	21	2	1	1	2	1	2	2	2	1
35	39	2	1	1	3	2	3	1	2	1

36	19	2	1	3	2	1	3	1	1	1
37	22	2	1	1	2	1	3	2	2	1
38	35	1	1	1	3	1	4	1	1	2
39	48	2	1	1	2	2	3	1	1	1
40	23	2	1	1	2	1	3	2	2	1
41	22	2	1	5	2	1	3	1	1	1
42	24	2	1	1	1	2	4	1	1	1
43	22	2	1	2	2	2	2	1	1	1
44	25	1	1	2	2	2	3	1	1	1
45	21	2	1	1	2	2	3	1	1	1
46	46	2	1	1	2	2	3	2	2	1
47	25	1	1	2	2	2	4	2	2	1
48	29	1	1	2	1	2	3	1	1	1
49	20	2	1	1	2	2	3	1	1	1
50	46	2	1	1	2	2	3	1	1	1
51	28	2	1	1	2	1	3	1	1	1
52	33	2	1	1	2	2	3	2	2	1
53	37	2	1	4	2	2	4	1	1	1
54	40	2	1	1	2	2	2	2	1	1
55	26	2	1	1	2	1	4	1	1	1
56	18	2	1	2	1	2	2	2	2	1
57	30	2	1	1	2	2	3	1	1	1
58	19	1	1	3	2	2	2	2	2	1
59	38	2	1	1	2	2	3	1	1	1
60	20	2	1	4	2	1	2	2	1	1
61	50	2	1	1	2	2	3	1	1	1
62	27	1	1	2	2	2	3	1	1	1
63	33	1	1	2	2	1	3	1	1	2
64	21	2	1	2	2	1	3	2	1	1
65	28	1	1	2	2	2	4	1	1	1
66	22	2	1	2	2	2	3	2	1	1
67	41	2	1	1	2	2	2	1	1	1
68	28	2	1	2	2	2	4	2	2	1
69	20	2	1	1	2	1	4	2	2	1
70	23	2	1	1	2	1	3	1	1	1
71	31	2	1	1	2	1	3	1	1	1

72	35	2	2	1	2	1	3	2	1	1
73	18	1	1	1	1	2	2	2	2	1
74	63	2	1	1	2	2	3	2	1	1
75	19	1	1	1	2	1	3	2	1	1
76	19	1	1	1	2	1	4	1	1	1
77	58	1	1	1	2	2	3	1	1	1
78	20	2	1	1	2	2	4	1	1	1
79	62	1	1	1	2	2	3	1	1	1
80	56	1	1	1	2	2	3	1	2	1
81	23	2	2	1	2	1	4	1	1	1
82	55	1	1	1	2	2	2	1	2	1
83	56	1	1	1	2	2	3	2	2	1
84	58	2	1	1	3	2	4	1	1	1
85	40	2	1	1	2	2	3	2	2	1
86	23	2	1	1	2	1	4	1	1	1
87	38	2	1	1	3	1	4	1	1	1
88	47	2	1	1	2	2	2	2	1	1
89	56	2	1	1	2	2	3	1	1	2
90	54	2	1	1	1	2	3	1	2	2
91	58	2	1	4	2	2	2	2	2	1
92	50	1	1	1	2	2	2	2	2	1
93	40	2	1	1	3	1	3	2	1	1
94	60	2	1	1	2	2	2	1	1	1
95	49	2	1	1	2	2	2	1	1	1
96	42	1	2	1	2	1	3	1	1	1
97	58	1	1	1	3	2	2	2	1	1
98	41	2	1	1	2	2	3	2	1	2
99	40	2	1	4	2	2	3	1	1	1
100	36	1	1	1	2	2	3	1	1	1
101	63	2	1	2	2	2	1	2	1	2
102	24	2	1	2	2	1	3	1	1	1
103	21	2	1	2	2	2	3	1	1	1
104	48	2	1	1	2	2	3	1	1	1
105	46	1	1	1	1	2	2	1	2	1
106	24	1	1	4	2	1	4	1	1	1
107	43	2	1	1	2	2	3	2	1	1

108	40	2	1	1	3	2	1	1	1	1
109	25	1	1	1	2	2	3	1	1	1
110	46	2	1	1	2	2	3	1	1	1
111	23	2	1	1	2	2	3	2	2	1
112	50	2	1	4	1	2	2	2	1	1
113	21	2	1	1	1	2	3	1	1	1
114	21	2	1	5	2	1	3	2	2	1
115	34	2	1	1	2	2	3	1	1	1
116	37	1	1	2	2	2	3	2	1	1
117	51	2	1	1	2	2	2	2	1	2
118	61	2	1	1	2	2	1	2	2	2
119	23	2	1	1	1	1	3	1	1	1
120	44	1	1	1	2	2	2	1	1	1
121	45	2	1	1	1	2	3	1	1	1
122	58	2	1	1	2	2	2	1	1	1
123	23	2	1	2	2	1	4	1	1	1
124	38	1	2	1	2	2	3	1	1	1
125	35	2	1	1	3	1	4	2	2	1
126	40	2	1	1	2	2	3	2	2	1
127	21	2	1	1	1	1	4	1	1	1
128	55	2	1	1	2	2	1	1	1	2
129	57	1	2	1	2	2	3	2	1	1
130	26	1	1	2	2	1	4	2	2	1
131	22	2	1	1	2	1	3	2	2	1
132	39	2	1	1	2	2	2	1	1	1
133	60	2	1	1	1	2	2	1	1	1
134	26	2	1	1	2	2	3	1	1	1
135	35	1	2	1	2	2	3	2	1	1
136	50	2	1	1	2	2	3	1	1	2
137	18	2	1	3	1	2	3	1	1	1
138	35	2	1	1	2	2	3	2	1	1
139	27	2	1	1	3	2	4	1	1	1
140	50	2	1	1	1	2	3	1	1	1
141	65	1	1	1	1	2	3	2	1	2
142	35	2	1	1	2	2	3	1	1	1
143	32	1	2	1	2	2	3	2	2	1

144	55	2	1	1	2	2	3	1	1	1
145	21	1	1	1	1	1	3	1	1	1
146	48	2	1	4	2	2	3	1	1	1
147	25	2	1	1	2	2	3	2	1	1
148	55	2	1	3	2	2	3	1	1	2
149	42	2	1	1	2	2	3	1	1	1
150	26	1	1	1	2	2	4	1	1	1
151	50	2	1	1	2	2	2	1	1	2
152	45	2	1	1	2	2	1	2	2	1
153	29	1	1	1	2	2	4	1	1	1
154	26	2	1	1	2	2	3	1	2	1
155	30	1	1	1	2	2	3	1	1	1
156	23	2	1	1	2	1	4	1	1	1
157	52	2	1	1	1	2	1	1	1	1
158	64	2	1	1	2	2	2	2	1	1
159	25	2	1	1	2	2	3	1	1	1
160	60	2	1	1	2	2	3	2	1	1
161	28	1	1	1	2	2	4	1	1	1
162	23	2	1	1	2	2	3	1	1	1
163	57	2	1	1	2	2	3	1	1	1
164	36	2	1	1	2	2	3	1	1	1
165	30	1	1	1	2	2	2	1	1	1
166	22	2	1	1	2	1	4	2	2	1
167	33	1	1	3	2	2	2	1	1	2
168	60	2	1	1	2	2	3	2	2	1
169	56	2	1	1	1	2	1	1	1	2
170	20	2	1	2	1	2	2	2	2	1
171	50	2	1	1	1	2	3	1	1	2
172	54	2	1	1	2	2	3	2	2	1
173	55	2	1	1	2	2	3	1	1	1
174	19	1	1	2	2	2	3	1	1	1
175	35	1	1	2	1	2	2	2	2	1
176	23	2	1	1	2	1	4	2	1	1
177	37	2	1	1	2	1	3	1	1	1
178	29	1	1	2	1	2	3	2	2	1
179	25	1	1	4	2	2	3	1	1	1

180	42	2	2	1	1	2	2	2	2	1
181	72	1	1	1	1	2	1	1	1	2
182	65	2	1	1	2	2	2	2	2	1
183	20	2	1	1	2	1	4	2	2	1
184	19	2	1	1	2	2	2	1	1	1
185	27	2	1	1	2	2	1	1	1	1
186	26	2	1	1	2	1	3	1	1	1
187	50	2	1	1	2	2	3	1	1	1
188	20	2	1	1	1	2	3	2	1	1
189	28	2	1	1	2	1	3	2	2	1
190	30	2	1	1	2	1	3	1	1	1
191	36	1	2	1	2	2	3	1	1	1
192	30	2	1	1	2	2	2	2	1	1
193	18	2	1	3	2	2	3	2	1	1
194	40	2	2	1	3	1	4	1	1	1
195	28	1	1	1	2	2	3	1	1	1
196	31	1	1	1	2	2	3	1	1	1
197	27	2	1	1	2	2	3	2	2	1
198	61	2	1	1	2	2	2	1	1	2
199	45	2	1	1	3	2	4	1	1	1
200	52	2	1	1	3	2	2	2	2	1
201	67	2	1	1	2	2	2	1	1	2
202	31	2	1	1	2	2	3	1	1	1
203	23	2	1	1	2	1	3	1	1	1
204	23	2	1	1	2	2	3	1	1	1
205	23	2	1	1	2	1	3	1	1	1
206	23	2	1	1	2	2	3	1	2	1
207	21	2	1	1	2	1	3	2	1	1
208	29	2	1	1	2	1	3	1	1	1
209	26	2	1	1	2	1	4	1	1	1
210	20	2	1	1	2	1	3	2	1	1
211	54	2	1	1	2	2	3	1	1	1
212	52	2	1	1	2	2	3	1	1	1
213	60	2	1	1	2	2	3	2	1	1
214	63	2	1	1	2	2	3	2	2	2
215	54	2	1	1	1	2	2	2	1	1

216	35	2	2	1	3	2	3	2	1	1
217	23	2	1	1	2	2	4	2	2	1
218	23	2	1	1	2	1	3	1	2	1
219	39	1	2	2	2	1	4	2	2	1
220	24	2	1	1	2	1	3	1	1	1
221	20	2	1	1	1	2	3	2	1	1
222	24	2	1	1	2	2	1	2	2	1
223	18	2	2	1	1	2	3	2	2	1
224	25	2	2	1	1	2	3	2	1	1
225	18	2	2	1	1	2	3	1	1	1
226	57	2	1	1	1	2	3	1	1	2
227	50	2	1	1	3	1	4	1	1	1
228	20	2	1	1	2	1	3	2	2	1
229	18	2	2	1	1	2	3	2	2	1
230	18	2	1	1	1	2	3	2	1	1
231	43	2	1	1	3	2	3	1	2	1
232	21	2	1	1	2	1	3	2	2	1
233	18	2	1	1	1	2	3	2	2	1
234	49	2	1	1	2	2	3	1	1	2
235	22	2	1	1	2	2	3	1	1	1
236	18	2	1	1	1	2	3	1	1	1
237	21	2	1	1	2	1	3	2	2	1
238	42	2	1	1	3	2	3	1	1	1
239	19	2	1	1	2	2	2	2	2	1
240	42	2	1	1	2	2	2	1	1	1
241	19	2	2	2	1	2	3	1	1	1
242	70	1	1	4	1	2	2	1	1	1
243	28	2	2	1	1	2	3	1	1	1
244	25	2	2	1	1	2	1	1	1	1
245	33	2	1	1	3	2	2	1	1	1
246	55	2	1	1	2	2	2	1	1	2
247	23	2	2	1	1	2	2	1	1	1
248	51	2	1	1	2	2	4	1	1	1
249	68	2	1	1	2	2	3	1	1	1
250	25	2	1	1	2	2	3	1	1	1
251	60	2	1	1	2	2	1	2	1	1

252	55	2	1	1	2	2	2	1	1	2
253	22	2	1	1	2	1	3	2	2	1
254	22	2	1	1	1	2	4	2	1	1
255	36	2	1	3	1	2	3	1	1	1
256	60	2	1	1	2	2	2	2	1	2
257	32	2	1	1	2	2	3	1	1	1
258	50	2	1	1	2	2	3	1	1	2
259	21	2	1	1	2	1	3	1	2	1
260	40	2	1	1	2	2	3	1	1	2
261	18	2	2	1	1	2	3	1	1	1
262	62	2	1	1	2	2	3	2	2	1
263	26	1	1	1	2	1	4	2	1	1
264	22	2	1	1	2	1	3	1	1	1
265	50	2	1	1	2	2	3	1	1	1
266	25	2	1	1	2	1	3	1	1	1
267	27	2	1	1	2	1	3	2	2	1
268	27	2	1	1	3	1	4	1	1	1
269	19	2	1	1	2	1	3	2	2	1
270	23	2	1	1	2	1	3	1	1	1
271	22	2	1	2	2	1	4	1	1	1
272	26	1	1	1	3	1	4	1	1	1
273	25	2	1	3	2	2	2	1	1	1
274	18	2	1	4	1	2	2	1	1	2
275	22	2	1	2	1	1	3	2	1	1
276	39	1	1	1	1	2	3	1	1	1
277	27	1	2	3	2	1	3	2	2	1
278	25	2	1	1	2	1	4	2	2	1
279	20	2	1	1	2	2	2	2	2	1
280	21	1	1	1	1	2	1	2	1	1
281	30	1	1	1	2	1	2	1	1	1
282	20	2	1	5	2	1	3	2	1	1
283	28	2	1	1	3	2	3	1	2	1
284	22	2	1	1	2	2	3	2	2	1
285	21	2	1	1	2	1	3	2	1	1
286	21	2	1	1	1	2	1	2	2	1
287	23	2	1	1	2	1	3	2	2	1

288	24	2	1	1	2	1	3	2	2	1
289	23	2	1	1	2	1	3	1	1	1
290	20	2	1	2	1	2	3	1	1	1
291	23	2	1	1	2	2	3	2	1	1
292	23	2	1	1	2	1	3	1	2	1
293	18	2	1	1	1	2	2	2	1	1
294	25	1	1	1	2	1	4	1	1	1
295	22	2	1	1	2	1	4	2	1	1
296	45	2	2	3	2	1	4	1	1	1
297	30	2	1	1	2	2	1	1	1	1
298	23	2	1	2	2	1	3	2	1	1
299	22	2	1	1	2	1	4	1	1	1
300	36	2	1	1	2	2	2	1	1	1
301	19	2	1	1	2	2	4	2	1	1
302	21	1	1	1	1	1	3	1	1	1
303	22	2	1	1	2	2	3	1	1	1
304	22	2	1	1	2	1	3	2	1	1
305	22	1	1	3	1	1	2	2	2	1
307	25	2	1	4	2	2	2	2	1	1
308	21	1	1	1	2	2	3	2	1	1
309	23	2	1	3	2	1	4	1	1	1
310	21	2	1	2	2	1	3	2	2	1
311	19	2	1	2	1	2	2	2	2	1
312	45	1	1	3	2	2	4	2	2	1
313	20	2	1	2	1	2	1	2	1	1
314	32	2	1	1	3	2	2	2	2	1
315	23	2	1	1	2	1	3	2	2	1
316	22	2	1	1	2	1	3	2	2	1
317	21	2	1	1	2	1	3	2	2	1
318	19	2	1	1	2	1	3	1	1	1
319	21	2	1	1	2	1	3	1	1	1
320	60	2	1	1	1	2	2	1	1	1
321	21	2	1	1	2	2	2	1	2	1
322	20	2	1	3	2	2	2	1	1	1
323	25	1	1	2	2	1	4	2	2	1
324	20	2	1	2	2	1	4	1	1	1

325	21	2	1	1	2	2	3	1	1	1
326	22	2	1	1	3	1	4	1	1	1
327	21	2	1	1	1	1	4	1	1	1
328	24	2	1	1	2	2	3	1	1	1
329	39	2	1	3	2	2	3	1	1	1
330	23	2	1	1	2	2	2	2	2	1
331	30	1	1	1	3	2	3	1	1	1
332	24	2	1	1	2	2	1	1	1	1
333	18	2	1	1	1	1	2	1	1	1
334	32	2	1	4	2	2	4	2	1	1
335	28	2	1	1	2	2	2	1	1	1
336	25	2	1	1	2	2	2	2	1	1
337	24	2	1	1	2	2	3	1	1	1
338	21	2	1	1	2	2	3	1	1	1
339	22	2	1	1	2	2	3	1	1	1
340	25	2	1	1	2	2	3	2	2	1
341	18	1	1	1	1	2	3	2	2	1
342	27	2	1	1	2	2	2	2	2	1
343	35	2	2	2	3	2	4	2	2	1
344	25	2	1	1	3	2	2	1	1	1
345	23	2	1	1	2	2	2	1	1	1
346	34	2	2	1	2	2	3	1	1	1
347	35	1	1	3	3	1	4	1	1	1
348	54	2	1	1	2	1	3	1	1	1
349	64	2	1	1	2	2	3	1	1	1
350	21	2	1	1	2	2	3	1	1	1
351	25	2	1	1	2	2	3	1	1	1
352	24	2	2	1	1	2	3	1	1	1
353	42	2	1	1	2	2	3	2	1	1
354	20	2	1	1	2	2	3	1	1	1
355	24	1	1	3	1	2	4	1	1	1
356	26	2	1	1	2	2	3	2	1	1
357	24	2	2	1	1	1	2	2	1	1
358	22	2	2	1	1	2	3	2	1	1
359	18	1	1	2	1	2	2	2	2	1
360	28	2	1	1	2	2	1	1	1	1

361	20	2	1	1	2	1	4	1	1	1
362	24	2	1	1	3	2	3	1	1	1
363	50	2	1	1	2	2	3	1	1	1
364	50	2	2	3	2	2	2	1	1	1
365	52	2	1	1	2	2	2	2	1	1
366	45	2	2	1	2	2	2	1	1	1
367	44	2	1	2	1	2	2	2	2	1
368	23	2	2	1	2	1	3	2	2	1
369	39	2	1	3	2	2	3	1	1	1
370	45	2	1	5	1	2	3	2	1	1
371	22	2	1	2	2	2	4	2	1	1
372	18	2	1	1	2	2	4	2	2	1
373	19	2	2	2	2	1	3	2	2	1
374	23	2	1	2	2	1	4	2	2	1
375	23	2	1	2	2	2	3	1	1	1
376	19	2	2	1	2	2	2	2	2	1
377	18	2	1	1	1	2	3	1	1	1
378	26	1	1	2	3	1	4	1	1	1
379	25	1	1	5	1	2	3	2	1	1
380	25	2	1	2	1	2	3	2	2	2
381	25	1	1	2	2	2	3	2	2	1
382	25	2	1	2	2	2	2	1	1	1
383	26	1	1	2	3	1	4	2	2	1
384	24	2	1	1	2	2	3	2	2	1
385	30	2	1	2	2	2	3	1	1	1
386	23	2	1	1	2	1	3	2	1	1
387	20	2	1	1	2	2	3	2	1	1
388	22	2	1	1	2	2	3	1	1	1
389	20	2	1	1	1	1	3	1	1	1
390	23	2	1	1	2	1	4	1	1	1
391	25	2	1	1	3	2	4	1	1	1
392	18	2	1	2	3	2	4	2	2	1
393	20	2	1	1	2	1	3	2	2	1
394	23	2	1	1	2	1	4	2	1	1
395	18	2	1	1	2	1	4	1	1	1
396	20	1	1	1	1	2	3	1	1	1

397	24	2	1	1	2	2	3	1	1	1
398	26	2	1	2	2	2	2	2	1	1
399	20	2	1	2	2	2	4	1	1	1
400	22	1	1	1	1	2	3	1	1	1
401	23	2	1	1	1	2	3	1	1	1
402	23	2	1	1	2	2	2	1	1	1
403	53	2	1	1	2	2	2	1	1	1
404	23	2	1	1	2	2	4	2	2	1
405	27	2	1	1	2	2	3	1	1	1
406	19	2	1	1	2	2	2	2	2	1
407	23	2	1	1	2	2	3	2	1	1
408	22	2	1	1	2	1	2	1	1	1
409	20	2	1	1	2	2	4	1	1	1
410	22	2	1	1	2	2	3	1	1	1
411	20	2	1	1	2	2	3	1	1	1
412	21	2	1	1	2	2	2	2	2	1
413	21	2	1	1	2	2	3	2	2	1
414	22	2	1	1	2	2	4	2	1	1
415	21	2	1	1	2	2	3	1	1	1
416	22	2	1	1	2	2	3	2	2	1
417	22	2	1	1	2	1	3	1	1	1
418	21	2	1	1	1	1	4	2	2	1
419	22	2	1	1	2	1	4	2	2	1
420	26	2	1	1	2	2	4	2	2	1
421	21	2	1	1	2	1	3	2	1	1
422	23	2	1	3	2	1	3	2	2	1
423	23	2	1	1	2	1	3	2	2	1
424	19	2	1	1	2	1	3	1	1	1
425	23	2	1	1	2	1	4	2	2	1
426	18	2	1	1	2	2	3	1	1	1
427	18	2	1	1	1	2	3	1	1	1
428	20	2	1	1	2	2	3	2	1	1
429	18	2	1	1	2	2	3	2	1	1
430	18	2	1	1	1	2	3	1	1	1
431	18	2	1	1	2	2	3	1	1	1
432	22	2	1	4	2	2	3	2	2	1

433	21	2	1	1	2	2	2	2	1	1
434	21	2	1	1	2	2	3	1	1	1
435	20	2	1	1	2	2	3	1	1	1
436	22	2	1	1	2	2	3	1	1	1
437	21	2	1	1	2	2	2	1	1	1
438	21	2	1	1	1	2	3	1	1	1
439	18	2	1	1	2	1	3	2	2	1
440	22	2	1	1	1	2	3	1	1	1
441	20	2	1	1	2	1	3	1	1	1
442	34	1	1	1	2	1	3	2	2	1

A2	A3	A4	B1	B2	B3	B4	B5	C1	C2	C3	C4
1	2	2	1	2	2	2	2	3	3	4	3
1	2	2	1	2	2	2	2	2	2	5	5
2	2	2	2	2	2	2	2	3	3	3	3
1	1	2	1	2	2	2	2	3	4	4	2
1	1	1	1	2	2	2	2	3	3	3	4
1	1	2	1	2	2	2	1	5	3	5	3
1	2	2	1	2	2	1	2	3	3	3	3
1	1	1	1	2	2	2	1	5	5	5	5
1	2	2	1	2	2	2	2	3	3	4	2
1	2	2	1	2	2	2	2	3	3	4	5
1	2	2	1	2	2	2	2	4	3	3	4
1	1	2	1	2	2	2	2	3	3	3	3
1	2	1	1	2	2	2	1	4	3	5	4
1	1	1	1	1	2	2	2	3	2	3	2
1	1	2	1	2	2	2	2	4	3	3	4
1	1	2	1	2	2	2	2	4	3	4	3
1	2	2	1	2	2	2	2	2	2	3	3
1	2	2	1	2	2	2	2	5	4	4	3
1	2	2	1	1	1	1	1	3	1	3	1
1	1	1	1	2	2	1	2	5	5	5	5
1	1	1	1	2	2	2	2	5	3	3	3
1	2	2	1	2	2	1	2	4	5	4	4
1	2	2	1	2	2	2	2	5	5	3	4
1	2	2	1	2	2	2	2	2	2	2	2
1	1	1	1	2	2	1	2	3	4	3	3
1	1	2	1	2	2	1	2	5	2	3	1
1	1	1	1	2	2	2	2	4	4	4	4
1	2	2	1	1	2	2	2	5	4	5	5
1	2	2	1	2	2	2	2	3	3	3	2
1	1	1	1	1	1	2	2	4	3	4	4
1	1	1	1	2	2	1	2	3	3	3	3
1	2	2	1	2	2	1	2	3	2	4	1
1	2	2	1	1	2	1	2	4	4	4	4
1	1	2	1	2	2	1	2	3	2	5	1
1	2	2	1	2	2	1	2	5	4	5	3

1	1	1	1	1	1	1	1	3	4	2	3
1	1	1	1	2	2	1	2	4	2	5	2
1	2	2	2	2	2	2	2	4	4	4	4
1	1	1	1	2	1	2	1	2	2	2	2
1	2	2	1	1	2	1	2	2	2	2	2
1	1	1	1	2	2	1	2	4	4	4	3
1	1	2	1	2	2	1	1	5	5	5	5
1	2	2	1	1	2	1	2	4	4	4	4
1	1	1	1	1	2	1	1	5	5	5	5
1	1	2	1	2	2	1	2	4	4	5	3
1	2	2	1	2	2	2	2	4	4	4	3
1	2	2	1	2	2	2	1	5	5	5	5
1	2	2	1	2	2	2	2	5	4	4	4
1	2	2	1	2	2	2	2	3	2	2	3
1	1	1	1	2	1	1	2	4	4	4	4
1	1	1	1	2	2	2	2	4	5	4	4
1	2	2	1	2	2	2	2	3	3	3	3
2	2	2	2	2	2	2	2	4	4	4	4
1	2	2	1	2	2	2	2	3	2	4	3
1	2	2	1	2	2	2	2	3	3	3	3
1	2	2	1	2	2	2	2	4	2	4	3
1	1	1	1	2	2	2	2	5	5	5	5
1	2	2	1	2	2	2	2	5	5	5	5
1	2	2	1	2	2	2	2	4	3	4	3
1	1	1	1	2	2	2	1	3	3	4	3
1	2	2	1	2	2	2	2	4	3	4	3
1	1	2	1	1	2	2	1	5	5	5	5
1	2	2	1	2	2	2	2	3	3	3	3
1	1	1	1	2	2	1	1	3	3	5	1
1	2	2	1	2	2	2	2	3	2	3	4
1	1	2	1	2	2	1	1	4	4	4	3
1	1	1	1	2	2	2	2	4	4	4	4
1	2	1	1	2	2	1	2	3	3	4	3
1	1	2	1	2	2	2	2	4	4	4	3
1	1	1	1	2	2	1	2	4	4	4	4
1	2	2	1	2	2	2	2	3	3	3	3

1	2	2	1	2	2	2	2	4	3	4	4
1	1	1	1	1	1	1	1	5	5	5	5
2	2	2	2	2	2	2	2	4	4	3	3
1	1	1	1	2	2	1	1	2	2	5	2
1	1	1	1	1	1	1	1	5	5	5	5
1	1	1	1	2	2	2	2	5	5	5	5
1	1	1	1	2	2	2	2	4	2	4	2
1	2	2	1	2	2	2	2	5	5	5	5
1	2	2	1	2	2	2	2	4	4	3	3
1	2	2	1	2	2	1	1	4	3	4	2
1	2	2	1	2	2	2	2	4	4	4	4
1	1	2	1	2	2	2	2	4	4	5	4
1	1	2	1	2	2	1	2	4	4	4	4
1	2	2	1	2	1	2	2	5	5	5	4
1	2	2	1	2	2	2	2	3	2	5	3
1	2	2	1	2	2	2	2	2	2	4	4
1	2	2	1	2	2	2	2	4	4	4	4
1	2	2	1	2	2	2	2	2	2	2	2
2	2	2	2	2	2	2	2	4	4	3	4
1	2	2	1	2	2	2	2	3	2	2	1
1	2	2	1	2	2	1	2	4	4	4	4
1	2	2	1	2	2	2	2	3	3	4	3
1	2	2	1	2	2	2	2	2	2	3	2
1	2	2	1	2	2	2	2	5	5	5	3
1	2	2	1	2	2	2	2	3	3	3	3
1	2	2	1	2	2	2	2	5	4	5	4
1	2	2	1	2	2	2	2	4	4	4	4
1	1	1	1	2	2	1	2	5	5	5	5
1	1	1	1	2	2	2	2	4	3	4	3
2	2	2	2	2	2	2	2	3	3	3	3
1	2	2	1	2	2	1	2	3	3	3	3
1	1	1	1	1	2	2	2	4	2	4	1
1	2	2	1	2	2	2	2	3	2	4	2
2	2	2	2	2	2	2	2	5	5	5	5
1	1	1	1	2	2	2	2	5	5	4	3
1	2	2	1	2	2	1	2	4	4	4	4

1	1	1	1	1	1	1	2	5	4	4	4
1	1	1	1	1	2	1	1	5	4	4	4
1	1	1	1	2	2	2	2	3	3	3	3
1	2	2	1	2	2	1	2	2	3	5	2
1	2	2	1	2	2	2	2	5	2	3	4
1	1	2	1	2	2	2	2	4	3	4	3
1	1	1	1	1	2	2	2	4	3	4	4
1	2	2	1	2	2	2	2	1	4	3	3
1	2	2	1	2	2	2	1	5	4	4	3
2	2	2	2	2	2	2	2	3	4	4	4
2	2	2	2	2	2	2	2	3	3	3	3
1	1	1	1	2	2	2	2	3	3	4	3
1	1	1	1	2	2	2	2	5	5	5	5
1	2	2	1	2	2	2	2	5	4	4	4
1	1	1	1	2	2	2	2	4	4	4	4
1	1	1	1	2	2	2	2	3	2	5	2
1	1	2	1	2	2	2	2	3	3	4	3
1	2	2	1	2	2	2	2	4	3	3	2
1	1	2	1	1	2	2	2	4	3	4	4
1	1	2	1	2	2	1	2	3	2	4	1
1	2	2	1	2	2	2	2	4	4	5	3
1	2	2	1	2	2	2	2	4	4	4	4
1	2	2	1	1	1	1	1	3	4	4	4
1	1	1	1	2	2	1	2	3	2	4	2
1	2	2	1	2	2	2	2	2	2	2	2
1	1	2	1	2	2	2	2	4	4	4	4
1	1	1	1	2	2	2	2	3	2	2	2
1	2	2	1	2	2	2	2	3	2	4	3
2	2	2	2	2	2	2	2	3	3	3	3
1	2	2	1	2	2	2	2	3	2	3	2
1	2	2	1	2	2	2	2	3	3	4	3
1	2	2	1	1	2	1	2	3	3	4	3
1	1	1	1	2	2	2	2	4	4	4	4
2	2	2	2	2	2	2	2	4	4	4	4
1	1	2	1	2	2	1	2	4	4	4	4
1	1	1	1	1	1	1	1	4	4	4	4

1	2	2	2	1	2	2	2	4	4	4	4
1	1	1	1	1	2	1	1	5	5	4	4
1	2	2	1	2	1	2	2	4	3	4	3
1	2	2	1	2	2	2	2	4	3	3	3
2	2	2	2	2	2	2	2	4	3	4	5
1	2	2	1	2	2	2	2	1	2	3	3
1	2	2	1	1	1	1	1	1	1	3	3
1	2	2	1	2	2	2	2	3	3	3	3
1	2	2	1	2	2	2	2	5	5	5	5
1	1	1	1	1	1	1	1	5	5	5	5
1	2	2	1	2	2	2	2	4	3	4	4
1	2	2	1	2	2	1	2	4	4	4	3
1	1	1	1	1	2	1	2	4	4	4	3
1	1	1	1	2	2	2	2	4	4	4	4
2	2	2	2	2	2	2	2	3	3	3	3
1	1	1	1	2	2	2	2	4	2	4	2
1	2	2	2	2	2	2	2	3	3	4	3
1	2	2	1	1	2	1	1	3	2	4	2
1	1	1	1	2	1	2	1	5	3	5	5
2	2	2	2	2	2	2	2	3	2	3	2
1	1	1	1	2	2	2	2	3	4	3	3
1	1	1	1	2	2	2	2	5	5	5	5
1	1	2	1	2	2	2	2	5	5	5	5
2	2	2	2	2	2	2	2	3	3	3	4
1	1	1	1	1	2	1	1	3	4	4	4
2	2	2	2	2	2	2	2	3	3	3	2
1	2	2	1	2	2	1	2	5	3	5	3
2	2	2	2	2	2	2	2	3	3	3	3
1	2	2	1	2	2	2	1	4	3	4	3
1	1	1	1	1	2	2	2	5	5	5	5
1	2	2	1	1	2	2	1	5	4	4	3
2	2	2	2	2	2	2	2	4	3	4	4
1	1	2	1	2	2	1	1	4	3	5	2
1	2	2	1	2	2	1	2	4	3	4	3
1	2	2	1	2	2	1	1	5	4	5	4
1	1	1	1	2	2	2	2	5	5	5	5

1	2	2	1	2	2	2	1	4	4	5	3
2	2	2	2	2	2	2	2	4	4	4	4
1	2	2	2	2	2	1	2	3	3	3	3
1	1	2	1	2	2	2	2	4	4	5	4
1	1	1	1	1	2	1	2	4	2	4	2
1	2	2	1	2	2	2	2	4	4	4	4
1	1	1	1	2	2	1	2	4	3	4	3
1	2	2	1	2	2	2	2	2	2	3	2
1	1	1	1	2	2	1	2	5	3	3	4
1	1	2	1	2	2	2	2	3	3	2	1
1	2	2	1	2	2	2	2	2	2	2	2
1	2	2	1	2	2	1	1	4	3	3	3
1	2	2	1	2	2	2	2	3	3	5	4
1	2	1	2	2	2	2	2	4	3	4	5
1	2	2	1	2	2	2	2	3	3	3	3
1	1	2	1	1	2	2	2	5	5	5	5
1	2	2	1	2	2	2	2	3	3	3	3
1	2	2	1	2	2	1	1	3	2	4	3
2	2	2	2	2	2	2	2	4	4	4	4
1	2	2	1	2	2	2	2	3	3	4	4
1	2	2	1	2	1	2	2	3	3	4	2
2	2	2	2	2	2	2	2	3	4	4	4
1	2	2	1	2	2	2	2	2	2	3	2
1	1	1	1	2	2	2	2	4	3	4	3
1	2	2	1	2	2	2	2	4	3	3	3
1	1	2	1	2	2	2	2	3	2	4	2
1	1	1	1	2	2	2	2	4	3	4	2
1	2	2	1	2	2	2	2	5	2	5	2
1	2	2	1	2	2	2	2	4	3	5	3
1	1	2	1	2	2	2	2	4	4	5	4
1	2	2	1	2	2	1	2	5	4	5	3
1	2	2	1	2	2	2	2	1	1	1	1
1	1	2	1	2	2	1	1	4	4	4	4
2	2	2	2	2	2	2	2	4	3	3	3
2	2	2	2	2	2	2	2	2	3	3	3
1	2	2	1	2	2	2	2	4	4	5	4

1	1	1	1	2	1	2	2	4	4	4	4
1	2	2	1	2	2	1	2	4	4	5	4
1	1	1	1	2	2	2	2	3	2	4	3
1	1	1	1	2	1	2	2	4	4	4	4
1	1	1	1	2	2	2	2	5	4	4	4
1	2	2	1	2	2	2	2	4	3	3	3
1	2	1	1	2	2	1	1	3	2	4	3
1	2	2	1	1	2	1	2	2	2	3	2
1	2	2	1	2	2	2	2	4	2	5	2
1	2	2	1	2	2	2	2	3	3	3	3
1	2	2	2	2	2	2	1	5	5	5	5
1	2	2	1	2	2	2	2	4	4	4	4
1	2	2	1	2	2	2	2	3	4	4	2
1	2	2	1	2	2	2	2	4	3	3	3
1	1	2	1	2	2	2	1	1	2	3	1
1	1	1	1	2	1	2	2	4	4	4	3
1	2	2	1	2	2	1	2	4	4	4	3
1	2	2	1	2	2	2	2	4	3	4	2
2	2	2	2	2	2	2	2	5	5	5	5
1	1	2	1	2	2	1	2	5	4	4	3
1	2	2	1	2	2	1	2	1	1	4	2
1	2	2	1	1	2	2	2	3	2	4	2
1	2	2	1	2	2	2	2	5	4	5	5
1	1	2	1	2	2	2	2	5	2	4	3
1	2	2	1	2	2	2	2	4	4	4	4
1	2	2	1	2	2	2	2	4	3	5	4
2	2	2	2	2	2	2	2	3	3	3	3
1	2	2	2	2	2	1	2	4	4	4	2
1	2	2	1	2	2	2	2	4	3	4	3
1	2	2	1	1	2	1	2	1	1	1	1
2	2	2	2	2	2	2	2	3	3	3	3
1	1	2	1	2	2	2	2	3	3	4	3
1	2	2	1	2	2	2	2	3	2	2	2
1	2	2	1	2	2	2	2	3	4	4	3
1	2	2	1	2	2	2	2	4	3	4	3
1	2	2	1	2	2	2	2	1	2	4	4

1	2	2	2	2	2	2	2	2	2	2	2
1	2	2	1	2	1	2	2	3	1	4	3
1	1	1	1	2	2	2	2	3	3	3	3
1	2	2	1	2	2	2	2	3	3	2	2
1	2	2	1	2	2	2	2	3	3	3	3
1	1	2	1	2	2	2	2	4	4	5	3
2	2	2	2	2	2	2	2	4	3	4	3
1	1	2	1	2	2	1	2	3	3	4	4
2	2	2	2	2	2	2	2	3	3	3	3
1	1	1	1	2	2	1	2	5	4	4	4
1	2	2	1	2	2	2	2	4	4	5	4
1	1	1	1	1	2	1	1	3	4	5	4
1	1	1	1	2	2	2	2	3	3	4	3
2	2	2	1	2	2	2	2	5	5	5	5
1	2	2	1	2	2	2	2	2	2	4	1
1	1	2	1	2	2	1	2	4	4	4	3
1	2	2	1	2	2	2	2	4	4	4	3
1	1	1	1	1	2	1	2	5	4	4	3
1	1	1	1	2	2	1	2	5	5	5	5
1	2	2	1	2	2	1	1	5	1	5	1
1	1	1	1	2	2	2	2	5	5	5	3
1	2	2	1	1	1	1	1	4	5	5	5
1	1	1	1	1	2	2	2	5	5	4	4
1	1	2	1	2	2	2	2	5	5	5	4
1	2	2	1	2	2	2	1	5	5	5	3
1	1	1	1	2	2	1	2	4	2	3	2
1	2	2	1	2	2	2	2	5	2	4	2
1	2	2	1	2	2	1	2	2	2	2	3
1	2	2	1	2	2	2	2	5	3	4	5
1	2	2	1	2	2	2	2	5	5	5	5
1	1	1	1	2	2	1	2	3	3	3	3
1	1	1	1	2	2	1	2	3	3	4	3
1	1	1	1	2	1	2	1	3	3	4	3
1	2	2	1	2	2	2	2	5	5	5	5
1	2	2	1	2	2	2	2	5	5	5	5
1	1	2	1	2	2	1	2	4	3	4	2

1	2	2	1	2	2	1	2	5	4	5	4
1	2	2	1	2	2	2	2	3	3	4	3
1	1	1	1	2	2	1	2	4	3	3	3
1	1	2	1	1	1	1	2	4	4	4	2
1	1	1	1	2	2	2	2	5	5	5	5
1	1	1	1	1	1	1	1	5	5	5	5
1	1	1	1	2	2	2	2	4	4	4	4
1	2	2	1	2	2	2	2	3	4	4	2
1	2	2	1	2	2	2	2	3	3	3	3
1	2	2	1	2	2	2	2	4	3	4	3
1	1	2	1	2	2	2	2	5	5	5	5
1	2	2	1	2	2	2	2	4	4	4	3
2	2	2	2	2	2	2	2	5	5	5	5
1	1	1	1	2	2	1	2	5	5	5	5
1	2	2	1	2	2	1	1	2	2	4	4
1	2	2	1	1	2	1	1	3	3	4	3
1	2	2	1	2	2	1	2	3	2	3	4
1	2	2	1	2	2	2	2	4	4	4	4
1	2	2	1	2	2	2	2	4	3	4	3
1	2	2	1	2	2	1	2	5	5	5	2
1	2	2	1	2	2	2	2	2	1	3	2
1	1	2	1	2	2	2	2	4	3	4	4
1	2	2	1	2	2	2	2	4	3	4	3
1	2	2	1	1	2	1	1	3	3	3	4
2	2	2	2	2	2	2	2	3	3	3	4
1	2	2	1	2	2	2	2	3	4	2	2
1	2	2	1	2	2	2	2	4	3	4	3
1	2	2	1	2	2	2	2	3	2	4	3
1	1	2	1	2	2	2	2	4	3	4	3
1	2	2	1	2	2	1	2	3	3	3	3
1	1	1	1	2	2	2	2	4	3	5	3
2	2	2	2	2	2	2	2	3	3	2	2
1	1	1	1	2	2	2	2	4	4	5	3
1	2	2	1	2	2	2	2	3	3	3	3
1	1	2	1	1	2	2	2	3	3	3	3
1	2	2	1	1	2	2	2	1	1	3	3

1	1	1	1	1	2	2	2	3	4	4	3
1	1	1	1	2	2	1	2	5	5	5	5
1	2	2	1	1	2	1	2	3	4	4	3
1	2	2	1	2	1	1	1	3	3	5	3
1	1	1	1	2	1	2	2	4	4	4	4
1	2	2	1	2	2	1	2	4	4	4	3
1	1	1	1	2	1	1	2	5	4	4	4
2	2	2	2	2	2	2	2	1	1	1	1
1	2	2	1	2	2	2	2	3	3	3	3
1	2	2	1	2	2	2	2	5	1	1	1
1	1	1	1	2	2	2	2	4	5	5	3
1	2	2	1	2	2	2	2	4	3	4	4
1	2	2	1	2	2	1	2	4	4	5	3
1	2	2	1	2	2	1	2	1	1	4	3
1	2	2	1	2	2	2	2	3	3	5	3
1	2	2	1	2	2	2	2	4	3	4	4
1	2	2	1	2	2	1	2	4	5	3	5
1	2	2	1	2	2	2	2	5	5	5	5
1	2	2	1	2	2	2	2	4	4	4	4
1	2	2	1	1	2	1	2	3	3	4	3
1	1	1	1	2	2	1	2	5	5	5	5
1	1	1	1	2	2	2	2	4	4	3	3
1	2	2	1	2	2	2	2	4	2	3	1
1	1	1	1	2	2	2	2	3	3	3	3
2	2	2	2	2	2	2	2	3	4	4	4
1	1	1	1	1	2	2	2	3	2	4	3
1	2	2	1	2	2	2	2	3	4	4	3
1	1	1	1	2	2	2	2	3	3	4	3
1	1	1	1	2	1	1	2	4	4	4	2
1	2	2	1	2	2	1	2	3	2	2	2
1	2	2	1	1	2	2	2	4	4	4	3
1	2	2	1	2	2	2	2	5	3	5	4
1	2	2	1	2	2	2	2	5	4	5	4
1	2	2	1	2	2	2	2	4	3	1	3
1	2	2	1	2	2	2	1	3	3	3	3
1	2	2	1	2	2	2	2	5	3	5	5

1	1	2	1	2	2	1	2	3	2	3	3
1	2	2	1	2	2	1	2	3	4	5	3
1	1	1	1	2	2	2	2	5	5	5	5
1	2	2	1	2	2	2	2	3	2	3	2
1	2	2	1	2	2	2	2	4	4	4	4
1	1	1	1	1	2	2	2	4	4	3	3
1	1	1	1	2	2	2	2	5	5	5	5
1	1	2	1	2	2	2	1	3	3	4	3
1	2	2	1	1	2	2	2	3	2	3	1
1	1	2	1	2	2	2	2	3	2	2	2
1	1	1	1	1	2	1	1	4	4	5	4
1	1	1	1	1	1	1	1	5	5	5	5
1	1	1	1	1	1	1	1	3	2	3	4
1	1	1	1	2	2	2	2	4	4	5	5
1	1	1	1	2	2	1	1	4	3	4	3
1	1	2	1	2	2	2	1	5	5	5	5
1	1	1	1	2	2	1	2	5	4	5	3
1	1	1	1	1	2	2	2	4	4	4	4
1	2	1	1	2	2	2	2	1	1	1	1
2	2	2	2	2	2	2	2	3	3	3	3
1	1	1	1	1	2	1	2	3	3	3	3
1	2	2	1	2	2	2	1	3	3	4	3
1	1	1	1	2	2	1	2	4	4	4	4
1	1	2	1	2	2	2	2	5	5	5	5
1	2	2	1	2	2	2	2	4	2	1	3
1	1	1	1	2	2	2	2	4	3	4	3
1	2	2	1	2	2	2	2	5	4	4	3
1	1	1	1	2	2	2	2	5	5	5	5
1	2	2	1	2	2	2	2	3	2	3	2
1	1	1	1	2	2	1	1	4	3	4	3
1	2	2	1	2	2	1	2	3	3	4	2
1	1	1	1	1	1	1	1	3	3	3	3
1	2	2	1	2	2	1	2	3	3	5	3
1	2	2	1	1	2	2	2	3	3	4	3
1	1	1	1	1	1	1	1	5	5	5	5
1	1	1	1	2	2	2	2	5	4	5	4

1	2	2	1	2	2	2	2	4	4	4	4
1	2	2	1	2	2	1	2	3	3	4	3
1	1	2	1	2	2	1	2	3	2	4	2
1	2	2	1	2	2	2	1	5	5	5	5
1	1	2	1	2	2	1	2	4	4	4	4
1	1	1	1	2	2	2	2	4	4	4	4
1	2	2	1	2	2	2	2	3	3	3	3
1	2	2	1	2	2	1	2	3	2	3	1
1	1	1	1	2	2	2	2	5	5	5	5
1	2	2	1	2	1	1	2	4	4	4	4
1	1	1	1	2	2	2	2	4	3	4	4
1	1	1	1	2	2	2	2	3	5	5	5
1	1	1	1	1	2	1	1	3	2	4	2
1	2	2	1	2	2	1	2	4	4	4	3
1	2	2	1	2	2	1	2	3	3	4	3
1	2	2	1	2	2	2	2	5	4	4	4
1	1	2	1	2	2	2	2	4	3	3	3
1	1	1	1	1	1	1	1	4	4	4	4
1	1	1	1	2	2	1	2	4	2	3	2
1	1	1	1	2	2	1	2	5	4	5	5
1	1	2	1	2	2	2	2	4	2	2	2
1	1	2	1	2	2	1	2	5	4	5	3
1	2	2	1	1	1	1	1	1	2	5	3
1	2	2	1	2	2	1	2	3	3	4	3
1	2	2	1	2	2	2	2	4	3	4	3
1	2	2	1	2	2	2	2	5	4	4	2
1	1	2	1	2	2	1	2	5	4	4	4
1	2	2	1	2	2	1	2	5	5	5	5
1	1	2	1	2	2	1	2	5	4	5	5
1	1	1	1	2	2	1	2	5	5	4	5
1	2	2	1	2	2	2	2	3	3	3	3
1	2	1	1	2	2	2	2	5	5	5	5
1	2	2	1	2	2	1	2	4	3	4	3
1	2	2	1	2	2	2	2	4	4	4	4
1	1	2	1	2	1	2	1	5	4	3	2
1	1	1	1	2	2	1	2	4	4	4	4

1	2	2	1	2	2	2	2	3	2	4	2
1	2	2	1	2	2	1	2	1	1	3	1
1	2	2	1	2	2	1	2	3	2	3	1
1	2	2	1	2	2	1	2	3	3	4	2
1	2	2	1	2	2	1	2	4	2	4	2
1	2	2	1	2	1	2	2	3	3	2	3
1	1	2	1	2	2	2	2	3	3	5	3
1	2	2	1	1	1	1	2	2	2	5	2
1	1	2	1	1	1	1	1	5	5	5	5
1	1	1	1	1	2	1	1	2	2	2	1

C5	C6	C7	C8	C9	C10	C11	C12	C13	C14
3	2	2	3	2	2	3	2	2	2
3	2	3	2	3	2	5	2	1	3
3	3	3	3	3	1	2	3	1	3
2	2	2	2	4	2	4	1	1	4
3	3	3	2	2	2	4	2	2	1
3	3	3	3	3	3	5	2	2	3
3	3	3	3	3	3	3	3	3	3
5	5	5	5	5	5	5	5	5	5
1	3	4	2	1	2	3	3	2	3
4	3	3	3	3	3	2	4	5	3
4	2	3	4	4	2	4	3	1	4
2	5	3	2	2	2	3	1	3	3
4	3	4	5	4	4	5	4	2	4
1	3	3	4	3	2	3	3	2	2
3	4	3	3	4	4	3	3	4	3
3	4	2	3	3	3	4	2	2	4
2	2	3	2	2	3	3	3	2	3
3	4	3	4	4	3	4	3	2	4
2	3	3	3	3	3	3	3	3	3
1	5	5	1	5	5	5	5	5	5
3	3	3	3	3	3	3	3	3	3
4	5	4	5	3	4	4	4	4	3
4	3	3	3	3	3	3	3	3	3
2	2	4	2	2	4	2	2	2	2
4	3	3	3	4	4	4	4	3	5
2	3	3	3	4	3	4	2	1	4
3	4	3	4	4	4	3	4	4	3
3	1	4	3	4	3	5	4	2	3
3	1	4	2	3	3	3	3	3	3
4	5	4	3	3	4	4	4	4	4
2	1	3	3	3	3	3	3	3	3
2	4	2	3	4	3	4	1	1	4
5	3	4	3	3	4	4	3	3	4
5	2	2	2	4	2	3	1	1	4
3	3	4	3	3	4	4	3	3	4

4	3	4	4	5	5	5	3	5	5
1	3	2	1	4	1	5	1	1	4
3	4	4	4	4	4	4	4	4	4
2	2	2	2	2	2	2	2	2	2
2	2	2	2	2	2	2	2	2	2
3	3	4	3	4	4	3	2	2	2
1	4	2	5	5	5	5	4	4	5
3	3	3	3	4	3	4	2	3	4
5	5	4	4	5	5	4	5	5	5
4	5	3	4	4	3	4	4	3	5
3	4	4	3	3	3	3	2	2	3
5	4	4	4	5	4	4	4	4	4
4	4	5	4	3	3	4	5	4	4
2	2	1	4	3	2	4	2	3	4
4	3	3	2	2	2	5	3	2	4
5	5	4	1	1	1	5	1	1	4
3	3	5	4	3	3	5	5	4	4
4	4	4	4	4	4	4	4	4	4
3	2	2	2	2	2	3	2	1	2
3	3	3	3	3	3	3	3	3	3
4	3	4	3	3	4	3	4	3	4
5	5	5	5	5	5	5	5	5	5
5	5	5	5	5	5	5	5	5	5
4	3	4	4	3	3	4	4	1	3
4	3	4	2	4	4	4	3	2	3
4	4	4	4	4	4	4	4	4	4
5	5	5	3	5	5	5	5	1	5
3	3	3	3	3	3	3	3	3	3
1	3	3	3	4	3	3	3	1	4
4	2	2	3	3	2	3	2	2	4
3	3	2	3	3	3	4	2	2	3
4	3	4	3	3	4	4	4	2	4
3	1	3	3	2	3	4	3	2	3
3	1	5	4	3	3	5	5	1	5
4	4	4	5	5	5	5	3	3	5
3	2	3	2	2	3	2	2	1	2

4	2	2	2	2	2	4	1	3	4
5	5	5	5	5	5	5	5	5	5
2	3	3	3	3	3	4	3	2	4
2	4	2	2	3	4	5	2	1	3
5	5	5	5	5	5	5	5	5	5
5	5	5	5	5	5	5	5	5	5
2	2	4	1	1	1	4	3	1	3
5	5	5	5	5	5	5	5	5	5
4	3	4	4	4	4	4	4	4	4
4	3	1	4	3	3	4	3	2	4
3	3	3	3	3	3	4	3	3	3
4	3	4	5	5	4	4	4	2	4
3	3	4	4	4	4	4	4	3	4
4	2	4	5	5	5	5	5	5	5
3	2	2	4	4	2	4	2	1	4
2	1	2	2	2	2	3	2	1	4
4	2	3	3	3	3	4	4	4	4
2	2	2	2	2	2	2	2	2	2
4	2	4	3	3	2	3	4	2	3
2	1	2	3	2	1	2	1	1	2
4	3	3	4	3	3	3	4	4	4
2	2	3	2	4	3	4	4	2	3
3	3	2	1	1	1	3	3	3	3
4	5	4	4	4	4	5	4	3	4
4	3	3	2	3	3	3	3	2	3
5	4	4	4	4	4	4	4	4	4
4	4	5	4	4	4	4	4	4	4
5	4	5	5	5	5	5	5	5	5
4	1	3	3	2	2	3	2	2	3
3	3	3	3	3	3	3	3	3	3
3	3	3	3	3	3	3	3	3	3
2	2	4	3	2	1	3	2	1	3
2	2	2	2	2	2	4	2	3	2
5	5	5	5	5	5	5	5	5	5
5	3	4	5	5	5	4	4	1	4
4	4	4	3	4	3	2	4	3	2

4	4	4	4	4	4	4	4	4	4
3	4	4	3	3	3	4	4	1	4
4	3	3	3	3	3	3	3	3	3
2	2	2	2	3	2	4	2	2	3
3	2	2	5	2	4	3	4	5	4
3	3	3	3	3	3	3	3	3	3
2	3	4	4	4	4	4	4	2	4
2	2	4	3	3	3	4	3	1	4
3	3	3	4	4	4	3	3	2	2
3	3	4	3	4	3	3	4	2	4
3	3	3	3	3	3	3	3	3	3
4	2	4	3	4	3	3	4	2	4
5	5	5	5	5	3	5	5	3	3
4	4	4	4	4	4	4	4	4	4
4	4	5	5	5	4	4	5	1	4
2	2	4	2	4	4	4	4	2	3
4	4	4	4	3	4	4	4	3	3
3	3	4	4	4	2	4	4	4	4
3	2	4	4	4	4	4	4	4	4
2	2	3	4	4	3	4	3	1	3
4	4	3	3	4	3	4	3	4	4
4	4	4	4	4	4	4	4	4	4
2	5	4	4	5	3	5	4	5	5
1	4	3	2	4	1	4	1	3	3
4	2	3	3	2	2	3	3	3	3
3	3	4	4	4	4	4	4	3	4
3	3	4	4	3	4	3	3	2	3
3	4	4	3	3	2	4	2	2	3
3	3	3	3	3	3	3	3	3	3
2	2	2	2	4	3	4	3	3	4
4	1	1	1	2	2	1	1	1	4
4	2	2	3	4	2	3	4	2	4
4	4	4	4	4	4	4	4	4	4
4	4	4	4	4	4	4	4	4	4
4	4	4	4	4	4	4	4	4	4
4	4	4	4	4	4	4	4	4	4

4	2	3	3	4	4	4	4	3	4
5	4	3	3	4	4	4	5	4	4
4	3	4	3	4	3	4	3	4	3
3	2	2	3	2	2	4	2	3	4
4	3	4	4	4	4	4	4	4	5
3	1	1	2	3	3	3	1	1	3
1	1	1	3	3	1	4	1	1	3
3	3	3	3	3	3	3	3	3	3
5	3	4	4	5	5	4	5	5	5
5	5	5	5	5	5	5	5	5	5
3	3	4	3	4	3	4	4	2	4
3	3	5	4	4	4	5	5	3	4
4	2	4	4	2	2	4	2	1	3
4	4	4	4	4	4	4	1	1	1
3	3	3	3	3	3	3	3	3	3
2	3	4	3	4	3	5	2	2	5
4	4	2	2	2	2	3	3	1	3
3	2	5	5	5	2	4	4	5	5
5	4	4	4	4	4	4	4	4	4
3	2	2	2	2	2	2	2	2	2
3	4	4	3	4	4	4	4	2	3
5	5	5	5	5	5	5	5	5	5
5	5	5	5	5	5	5	5	5	5
4	3	2	3	2	2	2	2	2	2
4	4	4	3	4	4	3	3	3	4
2	1	3	1	3	1	1	3	1	3
3	3	2	4	4	3	4	1	3	4
3	3	3	3	3	3	3	3	3	3
4	3	4	4	4	4	4	4	4	4
5	5	5	4	4	4	4	4	5	5
3	2	3	3	4	2	5	4	4	4
3	3	4	3	3	3	3	4	4	4
2	4	4	4	4	4	5	2	1	2
3	1	3	3	3	2	3	2	3	3
4	2	5	5	3	3	5	4	1	4
5	5	5	5	5	5	5	5	5	5

2	3	5	4	4	4	3	4	2	3
4	4	4	4	4	4	4	4	4	4
3	3	3	3	3	3	3	2	2	3
4	2	3	4	5	5	5	2	2	4
4	4	3	3	4	4	4	4	2	4
4	3	4	3	4	4	5	4	2	3
5	5	3	3	4	3	4	3	3	4
3	2	3	3	3	2	3	3	2	3
1	3	2	4	4	2	5	2	2	4
2	2	1	2	3	3	2	3	3	3
2	2	1	2	2	2	4	2	4	4
3	4	3	3	3	2	4	3	2	3
4	1	4	3	3	3	4	3	4	4
4	4	4	5	5	5	5	5	5	5
3	2	3	2	3	3	3	3	3	3
5	5	5	5	5	5	5	5	5	5
3	2	3	3	3	3	3	2	1	3
2	4	4	4	2	2	3	4	1	3
4	4	4	4	4	4	4	4	4	4
3	3	4	3	3	4	3	3	3	3
4	2	2	2	4	3	4	2	2	3
4	4	4	4	4	4	4	4	2	4
3	2	2	4	2	2	3	2	2	2
5	2	3	2	3	1	4	2	1	3
3	3	1	3	3	3	4	1	1	3
4	2	2	4	3	2	5	4	1	4
2	1	2	3	1	1	5	1	1	3
2	4	4	4	4	4	4	3	2	5
2	3	4	2	3	4	4	4	2	3
3	3	4	2	4	3	4	3	2	3
3	2	3	3	3	2	3	2	2	2
1	1	1	1	1	1	1	1	1	1
3	2	3	2	4	4	4	3	2	4
3	3	1	3	3	1	1	3	1	1
3	3	3	3	3	3	3	3	3	3
3	4	5	4	5	5	5	5	5	5

4	4	4	4	3	3	4	4	3	4
3	3	4	4	4	4	5	4	4	4
4	2	3	3	2	2	4	2	4	4
4	4	4	4	4	4	4	4	4	4
2	2	5	2	4	4	5	4	2	4
3	1	1	2	3	3	4	2	2	2
1	1	5	3	5	4	3	5	3	3
2	2	2	2	3	2	3	2	1	2
3	1	2	4	3	1	5	3	2	5
2	3	3	3	3	3	3	3	2	3
5	5	5	5	5	5	5	5	5	5
3	3	3	4	4	4	4	4	3	4
3	2	4	4	3	4	4	4	2	3
3	3	4	4	4	4	4	4	4	4
5	2	1	1	2	1	1	2	1	2
4	3	3	4	4	3	4	3	3	4
3	2	3	4	4	4	4	3	2	4
3	3	2	2	3	2	4	2	4	4
5	5	5	5	5	5	5	5	5	5
2	2	4	4	4	4	3	3	1	3
4	1	1	1	1	1	3	1	3	1
2	1	4	2	3	2	4	2	1	4
5	3	5	5	5	5	5	5	3	5
4	3	2	4	4	2	5	4	2	5
4	4	4	4	4	4	4	4	4	4
4	4	5	4	5	5	5	5	4	5
3	3	3	3	3	3	3	3	3	3
3	4	4	1	3	2	4	3	1	4
3	4	4	3	4	4	4	4	3	3
1	1	1	3	3	1	5	1	1	3
3	3	3	3	3	3	3	3	3	3
4	3	4	3	3	4	4	4	2	5
2	2	2	3	3	2	2	2	2	3
4	2	2	4	3	2	4	2	3	4
3	3	4	4	4	3	4	4	2	3
5	1	4	1	4	1	4	2	1	4

3	2	2	2	2	2	3	2	3	2
2	3	2	2	4	3	5	1	1	3
2	2	4	4	5	4	4	3	1	4
2	4	3	4	3	4	3	4	4	4
3	3	1	3	3	1	2	1	3	3
3	3	3	4	4	3	3	3	2	3
3	3	3	3	3	3	3	3	3	3
3	4	3	4	3	3	4	4	4	3
3	3	3	3	3	3	3	3	3	3
4	2	5	3	5	3	4	5	4	4
4	4	4	4	4	4	4	3	3	3
4	3	3	4	4	4	5	4	2	4
3	3	3	4	3	3	4	4	2	3
5	5	5	5	5	5	5	5	5	5
2	2	1	2	3	2	4	1	3	4
3	3	4	5	3	3	4	4	1	3
3	3	4	3	4	4	4	4	4	4
2	3	4	2	3	3	4	3	1	2
5	3	3	3	5	5	5	3	3	5
1	5	3	3	3	3	5	4	1	5
3	3	5	2	5	5	5	5	3	5
5	5	5	5	5	5	5	5	5	5
4	4	4	4	3	3	4	4	3	4
4	4	4	4	4	4	4	4	4	4
3	3	5	3	4	3	4	5	3	5
3	4	2	1	4	2	4	4	1	4
2	1	3	3	3	2	4	2	1	3
3	2	1	2	2	2	4	2	1	3
5	3	5	3	5	1	5	5	5	5
4	5	4	5	5	4	5	4	3	5
3	3	3	3	3	3	3	3	1	3
2	1	4	2	4	4	3	3	2	4
3	4	4	4	4	3	4	4	3	4
5	5	5	5	5	5	5	5	5	5
5	5	5	5	5	3	2	5	4	5
2	3	1	2	3	2	4	1	4	4

3	3	4	3	4	4	5	5	3	4
3	3	4	4	3	2	3	3	1	3
5	5	4	3	3	3	5	3	1	3
4	1	4	4	4	3	5	4	1	4
5	5	5	5	5	5	5	5	3	5
5	5	5	5	5	5	5	5	5	5
3	3	4	2	4	2	4	4	2	4
1	2	3	2	2	1	4	1	4	4
3	3	3	3	3	3	3	3	3	3
3	4	4	2	4	3	4	3	2	3
5	5	5	4	5	5	5	5	5	5
2	3	4	4	4	4	4	4	1	4
5	5	5	5	5	5	5	5	5	5
1	1	4	1	5	5	5	3	5	5
1	4	3	2	3	3	4	3	1	4
3	1	2	2	3	3	4	1	1	3
3	3	3	3	3	3	3	3	3	3
4	4	4	4	4	4	4	4	4	4
4	3	4	3	3	3	3	3	3	3
4	1	5	5	5	5	5	5	5	5
2	2	3	3	3	2	4	3	1	3
3	3	3	3	3	3	4	4	1	3
3	3	2	3	3	3	4	3	4	4
3	3	4	4	4	3	4	4	2	4
3	4	4	3	4	4	4	4	4	4
4	2	4	2	4	2	4	2	2	4
3	3	3	3	3	3	4	3	2	3
2	2	4	2	3	4	4	3	2	3
2	2	2	3	3	2	4	2	2	3
2	3	1	3	3	4	4	1	3	3
2	5	5	3	5	4	5	5	1	5
3	3	5	3	1	1	1	1	1	2
2	2	3	3	4	4	3	4	4	5
3	3	3	3	3	3	3	3	3	3
3	3	3	3	3	3	3	3	3	3
1	1	1	3	2	1	3	1	1	3

2	2	4	3	4	2	4	2	2	4
5	5	5	5	5	5	5	5	5	5
3	3	4	3	3	3	4	3	2	3
5	1	5	5	5	5	4	5	5	5
4	4	4	4	4	4	4	4	4	4
3	3	4	3	3	3	5	4	1	3
3	4	4	2	4	4	4	4	1	4
1	1	1	1	1	1	1	1	1	1
3	4	4	4	4	4	4	4	2	3
1	1	1	1	1	1	1	1	1	1
5	3	3	3	3	2	3	4	1	2
2	3	3	4	4	4	5	3	5	5
3	3	1	1	3	1	4	1	1	5
1	4	2	1	3	2	4	1	1	3
3	1	5	3	3	3	4	5	1	4
4	4	4	4	3	4	4	3	3	4
5	4	3	5	3	4	4	3	5	5
5	5	4	2	4	3	3	2	3	4
2	4	2	1	3	3	2	2	3	5
3	3	3	3	4	3	4	3	1	3
5	5	5	5	5	5	5	5	5	5
4	2	4	4	4	3	4	4	2	4
3	1	1	2	3	1	5	1	1	4
2	1	2	3	2	2	3	3	3	3
4	2	2	3	4	3	3	4	1	4
4	2	3	4	3	3	4	3	2	4
3	3	3	3	3	3	3	3	3	3
2	3	3	2	3	4	4	3	4	3
2	2	4	2	2	2	3	3	3	3
3	4	3	2	1	1	3	2	1	3
4	4	4	4	4	3	4	4	4	4
1	1	4	3	3	3	3	3	3	4
3	3	4	4	4	3	5	4	3	5
2	4	4	3	4	3	4	4	4	4
3	3	3	3	3	3	3	3	3	3
5	5	5	5	5	5	5	5	5	5

2	2	2	2	3	3	3	2	2	3
2	4	3	2	4	2	5	2	2	4
5	5	5	5	5	5	5	5	5	5
2	3	3	1	3	2	3	2	2	3
4	4	4	4	4	3	4	4	3	4
3	4	2	3	4	2	4	3	2	4
5	3	5	5	5	5	5	5	5	5
4	3	4	3	4	3	4	3	1	4
4	3	2	3	2	3	3	3	1	3
3	3	4	3	3	5	3	3	1	5
3	3	3	3	4	4	4	3	1	4
5	5	5	5	5	5	5	5	5	5
2	2	2	2	3	2	3	3	2	3
5	2	5	5	5	5	5	4	5	5
3	3	5	5	5	3	5	5	5	5
5	5	5	5	5	5	5	5	5	5
3	3	5	3	5	3	5	2	3	5
3	4	5	4	4	4	5	5	2	5
1	1	1	1	1	1	1	1	1	1
3	3	3	3	3	3	3	3	3	3
3	3	3	3	3	3	3	3	3	3
3	2	4	3	3	3	4	4	2	3
4	4	3	4	3	4	3	4	3	4
5	5	5	5	5	5	5	5	5	5
3	3	1	1	1	1	3	1	1	3
3	4	3	4	4	3	4	3	4	3
3	2	4	3	4	4	4	3	3	4
5	5	5	5	5	5	5	5	5	5
3	1	2	4	3	2	4	3	1	4
4	2	3	2	2	1	3	2	1	3
3	3	4	2	4	2	3	3	1	3
3	3	3	3	3	3	3	3	3	3
3	3	4	3	3	3	5	4	1	3
3	1	3	3	2	2	3	2	2	3
5	5	5	5	5	5	5	5	5	5
5	5	5	4	4	4	5	5	3	5

4	4	4	4	4	4	4	4	4	4
3	2	4	3	2	4	3	3	1	4
4	2	3	2	2	3	3	2	2	3
5	5	5	5	5	5	5	5	5	5
3	4	3	4	3	4	4	3	3	3
4	4	4	4	4	4	4	4	4	4
3	3	3	3	3	3	3	3	3	3
3	1	1	1	4	3	4	1	1	4
5	5	5	5	5	5	5	5	5	5
4	4	4	4	4	4	4	4	4	4
3	3	3	4	3	2	4	2	2	4
2	5	2	1	2	2	3	1	1	1
2	5	5	5	5	5	5	5	5	5
3	3	4	4	3	3	4	4	2	3
2	3	3	2	2	1	3	3	1	3
4	4	4	4	4	4	4	4	4	4
3	2	4	4	4	4	5	3	4	4
3	2	4	2	4	4	4	3	3	3
3	3	4	2	4	2	4	4	2	4
4	3	4	2	4	4	5	3	2	5
2	4	4	4	4	3	3	2	1	4
3	4	5	4	4	4	5	5	3	5
1	1	1	4	3	2	4	1	1	3
3	3	2	3	3	3	4	3	3	4
3	5	3	4	4	4	4	3	1	5
2	2	2	4	4	2	4	2	2	4
2	3	5	5	5	5	5	5	5	4
5	5	5	5	5	5	5	5	4	5
5	2	5	5	5	5	5	5	5	5
5	3	5	5	4	5	5	5	4	5
2	2	4	4	4	3	4	3	2	3
5	5	5	5	5	5	5	5	5	5
4	3	4	3	3	3	4	4	3	3
4	4	4	4	4	4	4	4	4	4
1	2	3	4	5	4	3	2	1	2
4	4	4	4	4	4	4	4	4	4

2	2	1	2	3	3	3	1	2	3
1	1	1	1	1	1	1	1	1	1
4	2	1	2	3	3	3	1	1	4
2	1	1	3	1	2	4	3	3	4
5	1	1	4	3	3	2	1	1	4
2	2	2	3	2	2	2	4	3	2
5	5	5	2	3	3	5	3	1	3
2	2	4	4	4	3	4	2	1	4
5	5	5	5	5	5	5	5	5	5
2	2	3	3	4	3	4	5	2	3
