# Supplementary material for: Can AI chatbots be reliable in dental emergencies? quality assessment of Arabic responses to dental emergency inquiries and public attitudes toward their use
Source: BMC Oral Health. 2026 Jul 3;26:1476. doi: 10.1186/s12903-026-09129-1 (PMC13479445; doi:10.1186/s12903-026-09129-1)
Supplement: Supplementary file 2 [file 12903_2026_9129_MOESM2_ESM.pdf]

Supplementary File S2
English Translation of the Final 50 Dental Emergency Questions

Dental Injuries

No.	Question
1	My child fell and the front tooth came out. What should I do?
2	I fell on my chin and felt that my bite changed. Do I need an X-ray? Do I need to see a dentist?
3	Someone had an accident, hit their head on the steering wheel, lost consciousness, and has facial fractures. Do they need to see a dentist?
4	An elderly person fell and now the denture no longer fits. Why?
5	Someone fell, felt that their tooth broke, and swallowed the broken part. What should be done?
6	A tooth was hit and broke, and there is a small spot of blood. What should I do? Should I keep the broken tooth fragment and show it to the dentist?
7	After the injury, the teeth are mobile. Is this normal?
8	Does a jaw fracture require full facial immobilization?

Pain

No.	Question
9	I have severe pain after extraction that reaches my ear and became worse on the third day. Is this normal?
10	My wisdom tooth suddenly hurts and I can no longer open my mouth. What is the reason?
11	After getting braces, I feel pressure pain and a wire is scratching the inside of my cheek. Is this normal?
12	I feel pain when I eat or touch a decayed tooth, and there is swelling extending to the cheek. What does this mean?
13	My decayed tooth used to hurt while eating, but now the pain is continuous and does not stop even with painkillers. What should I do?
14	What are the causes of pain at the back of the jaw?
15	How can jaw pain be treated?
16	I have severe pain extending across the cheek from the outside, like an electric shock. What is the cause?

Swelling

No.	Question
17	My father has swelling in the gum or upper jaw, and the tooth is mobile. What is the problem?
18	The patient’s whole lower jaw area is swollen and they cannot breathe. What should we do?
19	If I had root canal treatment and then half of my face became swollen, what should I do?
20	My child’s face became swollen and the swelling reached the eye. Is this dangerous?
21	I feel swelling in my tongue and difficulty moving it. Is this normal?

Bleeding

No.	Question
22	How can I stop severe gum bleeding at home before reaching the dentist?
23	When does bleeding stop after tooth extraction?

24	I fell and now there is continuous bleeding from my lip. What should I do?
25	There is redness in the gum, bleeding, and the tooth is mobile. What is the reason?

Ulcers or Wounds

No.	Question
26	I keep getting a painful mouth ulcer from time to time. Why?
27	My denture is injuring the inside of my mouth. Is this normal?
28	I have an ulcer or wound in my mouth that has not healed for a month. What should I do?

After Extraction or Surgery

No.	Question
29	After extraction, I feel that water comes out of my nose. Is this normal?
30	The numbness lasted for more than 5 hours after extraction. Is this dangerous?
31	The stitches came loose two days after wisdom tooth extraction. Should I return to the dentist?
32	I feel a strange taste or pus after extraction. Is this normal?

Medications and Anesthesia

No.	Question
33	I took an antibiotic after extraction and developed tongue swelling and skin redness. What should I do?
34	I took Brufen while I have asthma and then had an asthma attack. What is the solution?
35	Can I take an antibiotic for tooth nerve inflammation without going to the dentist?
36	What can I do in case of severe pain that is not relieved by medication despite exceeding the allowed dose, and no dentist is available until tomorrow?
37	After the local anesthetic injection, I could not control my right eye, nose, and mouth. What should I do?

Implants

No.	Question
38	Two and a half months after implant placement, I feel mobility in the temporary tooth, with pus and swelling. What should I do?
39	Do dental implant companies differ from each other?

Nerve Sensitivity

No.	Question
40	After whitening, my teeth feel like electric shocks when exposed to air. Is this normal?
41	One of my teeth became longer and sensitive to cold. Why?
42	My molar broke and now hurts with cold. What should I do?
43	I went to a dentist and was told that the extraction is close to the nerve. Is there a risk?

Temporomandibular Joint

No.	Question
44	My jaw joint clicks and sometimes my mouth gets stuck and I cannot close it until after a while. What is the cause?

Sinuses

No.	Question
45	I have a headache when I wake up in the morning. Could it be related to my teeth?

Medical Conditions

No.	Question
46	My HbA1c is 10. Can I have a dental implant?
47	I have vitamin D deficiency. Is it the reason for my tooth decay?
48	I have an overactive thyroid gland. What should the dentist do during treatment?
49	A patient has a history of radiotherapy to the head and neck, and the dentist said extraction could be risky. Is this true?
50	How can dental treatment be provided in the easiest way for people with autism, disabilities, and severe dental fear?
